# The influence of improved wheat and maize varieties on infant mortality in China

**DOI:** 10.1093/pnasnexus/pgaf048

**Published:** 2025-02-20

**Authors:** Xiaobing Wang, Xinyu Liu, Shi Min, Songqing Jin, Jikun Huang, Scott Rozelle, Jieyuan Feng, Boddupalli M Prasanna

**Affiliations:** China Center for Agricultural Policy (CCAP), School of Advanced Agricultural Sciences, Peking University, Beijing 100871, China; China Center for Agricultural Policy (CCAP), School of Advanced Agricultural Sciences, Peking University, Beijing 100871, China; China International Capital Corporation Global Institute, Beijing 100022, China; College of Economics and Management, Huazhong Agricultural University, Wuhan 430070, China; Department of Agricultural, Food and Resource Economics, Michigan State University, East Lansing, MI 48824-1039, USA; China Center for Agricultural Policy (CCAP), School of Advanced Agricultural Sciences, Peking University, Beijing 100871, China; Stanford Center on China's Economy and Institutions, Freeman Spogli Institute for International Studies, Stanford University, Stanford, CA 94305-6065, USA; Stanford Center on China's Economy and Institutions, Freeman Spogli Institute for International Studies, Stanford University, Stanford, CA 94305-6065, USA; International Maize and Wheat Improvement Center (CIMMYT), Nairobi 1041-00621, Kenya

**Keywords:** wheat, maize, high-yielding varieties, infant mortality, famine

## Abstract

The diffusion of high-yielding crop varieties has been a key driver for agricultural productivity. This study examines the relationship between the adoption of high-yielding crop varieties of two staple crops—wheat and maize—and infant mortality in rural China. Using data from 1954 to 1987, we find a significant reduction in infant mortality linked to high-yielding crop varieties diffusion, an association that remains robust even after excluding the Great Famine years. We investigate potential mechanisms driving this relationship, including increased grain production, improved infant nutrition, and changes in maternal characteristics. Additionally, our analysis unveils a spectrum of heterogeneous relationships between high-yielding crop varieties adoption and infant mortality across factors such as infant gender, maternal characteristics, and policy regulation. These findings reaffirm the positive and lasting benefits of dissemination of high-yielding crop varieties for human welfare and provide valuable policy insights for developing nations grappling with food and nutritional insecurity.

**Significance Statement**

This paper examines the relationship between the diffusion of high-yielding varieties (HYVs) and infant mortality in China, while controlling for health and income variables. The findings indicate that the diffusion of improved wheat and maize varieties is significantly associated with a reduction in infant mortality. Increased grain production and improved infant nutrition are identified as the key mechanisms driving this relationship. Additionally, this relationship is more pronounced for male infants. These results suggest that promoting HYVs could have positive implications for human welfare.

## Introduction

In China, the adoption and dissemination of high-yielding crop varieties (HYVs) predated the Green Revolution (GR), with the majority emerging in the 1970s and 1980s (1–4). By the 1970s, the intensified investment by the state in agricultural research and development, along with international collaborations, catalyzed the breeding of HYVs in China (5, 6). The extensive cultivation of wheat and maize serves as a noteworthy illustration of China's strides in improved varietal adoption, resulting in widespread acceptance of HYVs by Chinese farmers during the 1980s and 1990s (7, 8). Despite the consensus affirming the positive impact of HYVs on global food production, there is limited microlevel evidence on how the adoption of HYVs in wheat and maize influenced human welfare in China.

HYVs have emerged as pivotal drivers of aggregate food supply and were theorized to exert significant influence on economic growth (2, 9, 10). The existing literature predominately suggests that the adoption of HYVs has reduced infant mortality, with evidence from India and other developing countries (11, 12). For example, using a novel country-level indicator of modern crop variety (MV) diffusion and child individual data across 37 developing countries between 1961 and 2000, Goltz et al. (11) find that on average, MV diffusion reduced infant mortality by 2.4 to 5.3 percentage points. Similarly, Bharadwaj et al. (12) document that the adoption of HYVs in India since the GR led to significant declines in infant mortality, with greater benefits observed among underprivileged households and male infants.

The potential mechanisms underlying these reductions are hypothesized to include improved income and nutritional levels, though these pathways remain underexplored (13).^a^ However, a small strand of literature highlights potential adverse health impacts associated with the heavy reliance on agrochemical inputs accompanying HYVs adoption (14, 15). Beyond the empirical evidence, theoretical prediction of the impact of HYVs on health is less clear as it can be dependent upon household characteristics—specifically, whether a household is a net food seller or buyer (16). Increases in total production may depress food prices, reducing income for net food sellers, which could increase mortality rates (17). Overall, while the majority of evidence points to mortality declines, the literature reveals nuances in the magnitude and mechanisms of this relationship, as well as potential heterogeneities across socioeconomic status and contexts. In this study, we focus on infant mortality as a measure for infant welfare, recognizing it as a comprehensive indicator reflecting a nation's human development, poverty eradication efforts, and equitable distribution of economic growth across regions (18).

Utilizing a two-way fixed-effects model, our analysis reveals a strong positive association between the adoption of HYVs and reductions in infant mortality from 1954 to 1987 in rural China. We specifically concentrate on wheat and maize varieties, as the sample regions of our dataset mainly cover the northern Huang-Huai-Hai region, a key production area for these crops.^b^ Our analysis shows that the accumulative increase in the average weighted yield frontier of wheat and maize over the study period (1954–1987) is associated with a decrease in infant mortality of ∼0.14 percentage points in areas with 1 SD larger of the average crop suitability in rural China. Importantly, the positive relationship between the adoption of HYVs and reductions in infant mortality remains robust even after excluding the years of the Great Famine. To rule out potential mechanisms of concurrent health and income improvements, we conducted a set of supplementary estimates, which further support our findings. We also identified that the diffusion of HYVs is associated with the increased grain production and infants’ nutrition intake, which appear to be the two principal mechanisms driving the reduction in infant mortality. Finally, our investigation uncovers heterogeneous associations between HYVs adoption and infant mortality, varying by factors such as infant gender, maternal characteristics, and policy regulations.

In light of China's historical struggle with grain scarcity until the 1990s, we specifically use a dataset spanning from 1954 to 1987. We consider that this period presents a unique and critical timeframe to discern the implications of HYVs adoption on infant health outcomes. Notably, this timeframe encapsulates China's Great Famine, an unprecedented crisis starting from 1959 to 1961, surpassing the scale of all other documented famines (19). Despite commendable advancements in maternal and child health in subsequent years, contemporary challenges persist, underscored in part by China's sheer population size. As of 2022, the nation still contends with an infant mortality rate of 4.9‰, prompting an ongoing concerted effort to minimize this number.^c^ Particular focus should be on nutritional security in populations residing in remote areas, as well as in the densely populated urban regions grappling with inadequate medical coverage.

This study reinforces the existing body of evidence regarding the positive effect of HYVs diffusion on human welfare, specifically on infant mortality. First, while previous studies have identified positive health impacts resulting from the GR in developing nations (10, 12), the incorporation of evidence from China, one of the world's most populous developing countries, significantly strengthens the relevance of HYV-driven agricultural productivity growth and its impact on health outcomes. Secondly, our study deepens the understanding of the relationship between HYVs diffusion and infant mortality. After controlling for potential confounding factors related to health and income, we explored two mechanisms underlying these findings: increased grain production and improved nutritional intake. These factors emerged as key contributors to the decrease in infant mortality. Thirdly, the findings of this study hold important policy implications for low- and middle-income countries confronting scarcities in grain supply. Thus, increasing grain yields, enhancing the cultivation of HYVs, and promoting widespread HYVs adoption emerge as actions bearing positive implications for human welfare.

## The history of breeding and diffusion of wheat and maize varieties in China

The evolution of agriculture in China from the pre-People’s Republic of China (PRC) era (before 1949) through the postreform period illustrates a significant transition from traditional landraces to high-yielding selectively bred crop varieties. Before the establishment of the PRC in 1949, crop cultivation was primarily overseen by various universities, missionaries from foreign agricultural agencies, and the Chinese National Agricultural Bureau. During this initial phase, farmers predominately relied on landraces, or locally adapted varieties of cultivated plant species (20). The transitional period of the 1950s and 1960s witnessed a notable evolution as specific landraces, newly developed crop varieties, and improved materials developed domestically and abroad gradually supplanted traditional landraces (21, 22). By the late 1970s, wheat varieties selectively bred for characteristics such as high-yield potential, resistance to rust, and early maturity prevailed (3). The selection of superior high-yielding maize germplasm is one of the most important decisions made in crop cultivation, with profound implications on agricultural productivity during the 1970s (23, 24). Following rural reforms initiated in 1978, farmers gained greater autonomy in selecting which crop varieties they wished to cultivate (4). This shift led to a surge in the number of crop varieties released and grown during the 1980s and 1990s (25). Consequently, the postreform era witnessed an increased turnover of crop varieties, accompanied by a discernible decline in the utilization of wheat and maize varieties which were the predominant crops in the preceding two decades (26).

While China's own domestic system deserves much of the credit, the nation's agricultural sector has greatly benefited from collaborations with international research institutions, particularly in the adoption and development of HYVs. China's scientific and agronomic communities forged close collaborations with prominent international institutions, such as the Consultative Group on International Agricultural Research (CGIAR) centers and other international research centers, in the development of HYVs (27). In the context of wheat cultivation, the first improved varieties derived from CIMMYT (International Center for Maize and Wheat Improvement) were introduced in China from Pakistan in the late 1960s (5). Additionally, several external sources of germplasm from countries such as Italy (Villa Glori, Mentana), Chile (Orofen), the USA (Minn2761, Triumph, Early Premium), and Australia (Quality) significantly contributed to the genetic lineage of advanced Chinese wheat lines (28). By the 1970s, China introduced various semidwarf CIMMYT varieties, including Yecora F70 and Tanori F71 (7).

One example of the interactions between China and the rest of the world is the way that breeders in China successfully integrated international germplasm with local wheat varieties through hybridization, resulting in resilient strains adapted to regional conditions. The exotic varieties faced challenges, such as susceptibility to rust, premature sprouting, and later maturity compared with the local varieties in China. In response, Chinese breeders initiated hybridization between CIMMYT cultivars and indigenous wheat varieties, resulting in the development of strains such as Kehong16, Ningchun4, and Jinan17 (6). These varieties proved well-suited to the diverse conditions of southwestern and southern China. Over half of the wheat varieties released between 1950 and 1986 were the outcome of hybridizations between imported strains and Chinese landraces or their derived variations (7).

Apart from high-yielding wheat varieties, the adaptation of maize in China also highlights the transformative role of the research/breeding system in China. Maize, originating from Mexico, embarked on a transformative journey to China in the early 16th century via three distinct routes. ^d^ After nearly five centuries of adaptation, a rich tapestry of maize landraces evolved, characterized by diverse ecotypes and localized variations (29). In the 1920s and 1930s, China witnessed the adoption of tens of thousands of dent and flint maize landraces with favorable traits (30). These included indigenous varieties, such as Huobai, as well as those imported from the United States (e.g. the White Crane, Golden Queen, and Silver Queen) and Italy (e.g. Italy white) (31). Yet, China's average maize yield remained modest, reaching 64.1 kg/mu (961.5 kg/ha) by 1949 (32).

Beyond traditional varieties, the introduction of hybrid maize breeding in China since 1949 has catalyzed a paradigm shift, leading to the extensive adoption of high-yielding hybrid varieties over the past several decades. Beginning in 1949, maize breeding underwent a transformation with the initiation of hybrid breeding. This transformative period saw the emergence of Fangza2, released in 1949, which stood as the earliest widespread hybrid maize variety across the country (33). Furthermore, China's scientists pioneered the development of the maize inbred line Zi330, which became an elite inbred line in the late 1960s, facilitating creation of over 40 maize hybrids (34). The use of hybrid breeding contributed to the increase of maize yields in the postrevolution China (33). By the late 1960s, China's average maize yield climbed to 115 kg/mu (1,725 kg/ha) (8).

China's own research system, however, not only borrowed new technologies from outside of China, but also produced its own breakthroughs. For example, the strategic development and promotion of single-cross maize hybrids in the mid-1960s marked a progressive phase in China's maize breeding program. In the mid-1960s, China's scientists began developing and promoting single-cross maize hybrids. A series of single-cross maize hybrids were released, bearing distinctive names such as Xindan1, Baidan1, Baidan2, and Jidan101 (35). In particular, the breeding and promotion of Xindan1 marked a new stage of maize breeding in China characterized by the prevalence of single-cross hybrids (36). China also emerged as one of the pioneering nations to extensively implement single-cross hybrid maize breeding (37).

And there are more examples of China's ability to move beyond the simple introduction of foreign technologies; the selection of superior inbred lines from maize germplasm is one of the most important decisions made in crop cultivation, with profound implications on agricultural productivity from 1970s (23). Exchange of elite maize germplasm played a pivotal role in driving the growth of China's maize yields during the 1970s (24). Despite the absence of diplomatic relations between China and the United States, China's scientists nonetheless initiated germplasm exchange with American scientists (38). An exemplary outcome of this international collaboration was the introduction of Mo17 from the United States to China in 1974, which was then widely integrated into China's maize breeding efforts (39). Consequently, by the advent of the 1980s, China's average maize yield surged to 205 kg/mu (3,075 kg/ha) (40).

In summary, innovations in wheat and maize breeding have been a cornerstone in the substantial enhancement of China's crop yield from the early 1950s to the early 1980s. This transformative period witnessed an extraordinary surge, with the average trial yield of wheat and maize varieties escalating by an impressive 186 and 249%, respectively. The magnitude of this increase not only played a pivotal role in fortifying China's food security but also served as a catalyst for population growth (41). To a certain degree, advancements in breeding high-yielding crops also played a role in fostering social stability and propelling economic growth in the postrevolution China (17).

## Data and variables

### The infant mortality dataset

**Fig. 1.**
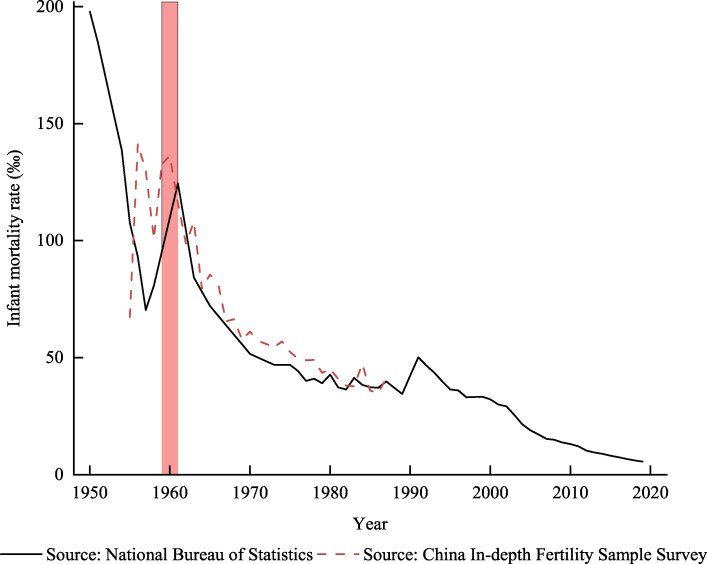
The infant mortality rate from 1950 to 2020. The shadowed area represents the famine years.

The infant mortality dataset was retrieved from China's In-depth Fertility Sample Surveys (henceforth referred to as “fertility surveys”) of 1985 and 1987, which were conducted by the China Population Information and Research Centre of the National Bureau of Statistics. In April of 1985, the first phase of the survey was implemented in Shanghai municipality and Hebei and Shaanxi provinces. The second fertility survey, following the same framework, was conducted in April of 1987 in Beijing municipality and Liaoning, Shandong, Guangdong, Guizhou, and Gansu provinces (see Fig. S1 of the Supplementary material).^e^ Employing a stratified, phased, and probability-proportional sampling approach, the fertility surveys identified sampled cities (counties), towns, and communities (villages). In the last stage of random sampling, the equal probability (self-weighting) method was used to select households, from which all qualified women were interviewed by survey enumerators (see Appendix S1.1 of the Supplementary material for more details).^f^ Figure 1 presents the trends of infant mortality from 1950 to 2019 in China, showing a remarkable decline of infant mortality in China from 200‰ in 1950 to 5.6‰ in 2019. The infant mortality rate peaked during the period from 1959 to 1961, coinciding with the Great Famine. The solid and dashed lines represent infant mortality rates sourced from national statistics or calculated from the survey data, respectively. The coherence between the fertility survey-based infant mortality trend and official statistics implies the survey's representation of women within childbearing age and their fertility histories. The descriptive statistics presented in Table 1 show that based on the sample of 65,362 infants in rural area, the average infant mortality rate was 63‰ over 1954–1987. To address the concern that migration might bias our estimates, the main results are based solely on the subsample of individuals with rural *hukou.*^g^

**Table 1.** Descriptive statistics of rural sample.

Variable definition	Obs	Mean	SD	Min.	Max.
Dependent variable					
Whether infant died	65,362	0.063	0.243	0	1
Independent variable					
Lnyield_wheat	65,362	6.062	0.212	5.387	6.516
Lnyield_maize	65,362	6.263	0.311	5.170	6.686
Lnyield_aver	65,362	6.190	0.224	5.392	6.614
Num_wheat	65,362	4.098	3.705	0	17
Num_maize	65,362	1.504	2.219	0	14
Amount	65,362	5.603	4.808	0	22
Suit_wheat	65,362	0.001	0.009	−0.022	0.022
Suit_maize	65,362	0.000	0.009	−0.014	0.024
Suit_aver	65,362	0.001	0.007	−0.013	0.020
Control variable					
Individual level					
Child male	65,362	0.524	0.499	0	1
Birth order number	65,362	2.476	1.602	1	15
Child multiple	65,362	0.009	0.092	0	1
Boy before	65,362	0.456	0.498	0	1
Birth weight	65,362	6.426	1.082	4	9
Mother edu	65,362	0.001	0.031	0	1
Mother work	65,362	0.694	0.461	0	1
Mother age at childbirth	65,362	25.594	4.500	16	47
Father age at childbirth	65,362	28.201	5.339	16	60
Wealth_index	65,362	−0.174	0.878	−1.770	29.810
Urban	65,362	0.000	0.000	0	0
Place_assist	65,362	0.241	0.428	0	1
Pregnant_check	65,362	0.089	0.285	0	1
County level					
Grain production	145	0.294	0.222	0.001	1.471
Teacher number	145	1683.434	2120.049	81	17,198
School number	145	341.200	278.734	16	2193
GDP	145	0.050	0.079	0.002	0.418
Industry ratio	145	0.211	0.171	0.014	0.950
Service ratio	145	0.216	0.144	0.017	0.830
Family savings	145	0.030	0.127	0.000	0.998
Fixed asset	145	0.011	0.050	0.000	0.411
Government expenditure	145	0.100	0.119	0.004	0.565
Population density	145	0.195	0.729	0.001	4.689
Average temperature	65,362	11.359	3.673	−18	29.6
Average precipitation	65,362	522.757	263.162	0	3270
Province level					
Cultivated area	65,362	1.094	0.660	0.074	3.126
Quota	65,362	9.052	11.387	−14.680	47.680
Ln_gdp	65,362	5.705	0.761	4.522	8.246

The data sources from which the variables come from are China In-depth Fertility Sample Surveys (1985 and 1987) and HYVs dataset. The county-level statistics are hand collected by ourselves from *Wanfang Local Chronicles Knowledge Service Platform* and *China Economic and Social Big Data Research Platform*, which are only observed in the baseline. The observations of the county-level variables are equal to the number of the county.

### HYVs dataset

**Fig. 2.**
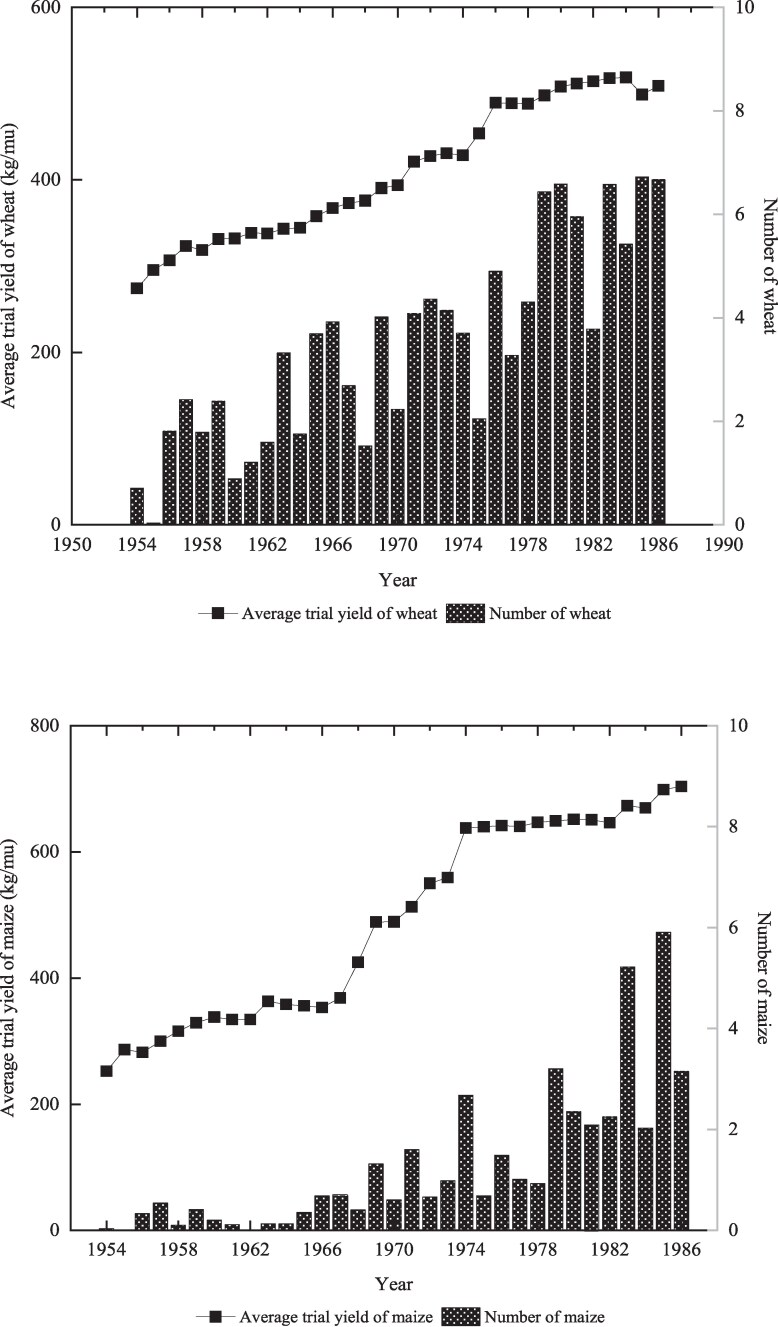
The average trial yield and numbers of diffused HYVs over 1954–1987. A) Wheat. B) Maize.

In this study, HYVs are defined as certified improved varieties of wheat and maize adopted at the province level. The data were compiled from multiple sources, including “Chinese Crop Varieties (1962–1993),” “Crops Varieties and Their Genealogy in China,” and “Variety Improvement and Genealogy Analysis in China.”^h^ Figure 2 shows that the trial yield of wheat varieties increased by more than 86% from 1954 to 1987, reaching 509 kg/mu (7,635 kg/ha) in 1987. Similarly, the trial yield of maize rose by over 149%, from 282 kg/mu (4,230 kg/ha) in 1954 to 704 kg/mu (10,560 kg/ha) in 1987. The figure also illustrates that the number of wheat and maize cultivars diffused in China increased substantially from 1954 to 1987. During that period, the assortment of wheat varieties expanded from 1 to ∼7, while the number of maize cultivars surged to 4 in 1987, a notable increase from the mere 1 introduced in 1954.

## Methods

To examine the relationship between HYVs adoption and infant mortality, we used ordinary least squares (OLS) as presented in the equations below:


(1) $$\mathrm{infan}t_{\mathrm{icsy}}=\alpha +\beta \;\mathrm{Suitabilit}y_{c}\times \mathrm{HY}V_{s,y-3∼y-1}+\gamma X_{\mathrm{icsy}}+\sigma Z_{c}\times \mathrm{Time}+\eta_{y}+\delta_{p}+\zeta_{\mathrm{sy}}+\varepsilon_{\mathrm{icsy}\;}.$$

(2) $$i\mathrm{nfan}t_{\mathrm{icsy}}=a+\beta \;\mathrm{Suitabilit}y_{c}\times \mathrm{HY}V_{s,y-3∼y-1}+\gamma X_{\mathrm{icsy}}+\sigma Z_{c}\times \mathrm{Time}+\eta_{y}+\delta_{p}+\zeta_{s}\times y\;+\varepsilon_{\mathrm{icsy}}.$$

Here, $\mathrm{infan}t_{\mathrm{icsy}}$ is an indicator equal to 1 if infant i born in year y at county c, province s died in the first 12 months after birth, 0 otherwise. $\mathrm{Suitabilit}y_{c}$ represents the land suitability of maize or wheat at the county c, and $\mathrm{HY}V_{s,y-3∼y-1}$ represents the moving average of trial yield frontier of newly promoted HYVs or number of newly promoted HYVs in province s from year (y−1) to year (y−3).^i^ We used the lag average form to allow for the delayed effects of HYVs adoption to manifest and to reduce the possible reverse causality concern. We also used the moving average of 2-year lag or 5-year lag to 1-year lag as a robustness check (see Tables S8 and S9 for reference). Following the method used by Nunn and Qian (13) and Macchiavello and Morjaria (42), we interacted the provincial level HYVs with the county-level crop suitability, $\mathrm{Suitabilit}y_{c}\times \mathrm{HY}V_{s,y-3∼y-1}$, to account for the fact that the agro-ecological conditions required for the cultivation of each crop are also correlated with its potential yield. This interaction term captures the accumulative temporal and geographical variations of HYVs adoption at the county level. The coefficient of interest *α* was hypothesized to be negative. While the trial yield frontiers of wheat and maize cultivars serve as our main measure of HYVs adoption, we also considered the number of newly promoted HYVs as a robustness check. This choice was made because the trial yield frontier displays an upward trend over the study period, providing a more accurate measure of crop promotion. In contrast, the number of promoted HYVs varies based on the actual promotion circumstances in each year.

To identify the association between HYVs adoption and infant mortality, we also controlled for other confounding factors $\left(X_{\mathrm{icsy}}\right)$ that may correlate with both HYVs adoption and infant mortality. The vector of control variables includes the characteristics of the infant, the parents, and regional-level traits. The infant characteristics include a dummy variable for the infant's gender, the infant's birth order, a dummy variable indicating whether the infant was part of a multiple birth, a dummy variable for whether a boy was born before the infant, and the birth weight of the infant. The mother's characteristics include a dummy variable for whether the mother finished high school and above, a dummy variable for the mother's working status, the age of the mother when delivering the infant, and a dummy variable indicating whether the mother resides in an urban or a rural area. The father’ age when the infant was born was also included as a control. Additionally, we also controlled for the quota, the cultivated area per capita, and the gross regional production per capita at the province level.

One of the challenges in the identification strategy is that other things are happening at the county level that are correlated with the adoption of HYVs and also with the outcome variable. To further address the potential endogeneity concerns that are associated with omitted factors correlated with both the treatment status and the outcome variables, we include a rich set of interaction terms between pre-1954 county-level characteristics and linear cohort trends $\left(Z_{c}\times \mathrm{Time}\right)$, following Hoynes et al. (43). These interactions account for potential time-varying unobservables at the county level that could influence both the treatment variable and infant survival probabilities. The county-level characteristics we use are consistent with prior studies (44, 45) and encompass multiple dimensions, including *Food Security* (Grain production), *Economic Development* (gross domestic product [GDP], industry ratio, service ratio, population density)*, Educational Level* (e.g. teacher number and school number), and *Financial System* (e.g. family savings, fixed asset, and government expenditure). The definition and descriptive statistics of theses variables are presented in Table S1 and Table 1, respectively.

Finally, we included several important sets of fixed effects. The first set is prefecture fixed effects $\delta_{p}$, which control for all time-invariant characteristics of the prefecture. For instance, if a prefecture had a lower level of HYVs adoption as well as higher infant mortality due to unsuitable land and extreme weather, controlling for the overall land and local weather endowment of the prefecture allows us to better gauge the correlation between HYVs adoption and infant mortality. The second set of fixed effects is birth-year fixed effects $\eta_{y}$, which account for any time-specific shocks, such as economic recessions, that affected all districts equally in the birth year. Apart from the above fixed effects, we further included either province-year fixed effects $\zeta_{\mathrm{sy}}$ in Eq. (1) or province-specific linear time trends $\zeta_{s}\times y$ in Eq. (2) as robustness checks. The specification with province-by-year fixed effects allowed us to account for any annual pattern in birth outcomes that may differ across provinces and were not captured by observable controls included. The second approach, using province-specific linear trends, accounts for possible unobserved trend variables that may vary linearly by birth cohorts in each province. In all regression models, the SEs, $\varepsilon_{\mathrm{icsy}\;}$, are clustered by county to account for within-county correlation over time.

Since the improvement in public health is a significant factor contributing to the decrease in infant mortality, we further controlled for a set of variables related to the prenatal health accessibility to assess the robustness of the effect of HYVs adoption on infant mortality. Specifically, prenatal healthcare variables include dummies indicating whether the mother underwent any pregnancy examination by a doctor in the 6 months prior to delivery and whether the child was delivered at home, hospital, or clinic assisted by a doctor, nurse, or midwife (1 = yes; 0 = otherwise). Given the absence of an income variable to account for the wealth mechanisms, we computed a standard index using the households’ possessions to gauge households’ wealth condition.^j^ We employed principal component analysis for this purpose, following the approach suggested by von de Goltz et al. (11). Additionally, we controlled for temperature and precipitation at the county level to isolate the impacts of exposure to the HYVs adoption from the impacts of weather in the infant's birth year. For further details on variable definitions, refer to Table S1 of the Supplementary material.^k^

In addition, we may be still concerned that certain families with infants may be migrated to reside in areas where HYVs are adopted due to the demographic and districts characteristics. This could potentially make them more exposed to the benefits of HYVs diffusion and bias our results. During the study period of China, it is highly unlikely for rural farmers to self-select to reside in certain areas because migration was largely constrained by the Chinese *hukou* system (45). One possible exception for individuals to reside in different areas was through their marriages. However, most of the rural women (or men) are married to husbands (or wives) who were born in nearby villages or towns, especially during the study period (46).^l^ To mitigate potential biases from migration, we restricted our main analysis to individuals with rural *hukou*, as these individuals were less likely to migrate between regions during the period of study.

## Results

### Main results

We begin this section by presenting the OLS estimates of Eqs. (1) and (2), along with their respective variants. It is noteworthy that the primary difference between the two equations lies in the substitution of the province-by-year fixed effects in Eq. (1) with the province-specific linear time trend in Eq. (2). All regression specifications discussed in this section incorporate controls for birth-year fixed effects, prefecture fixed effects, income effects, prenatal healthcare effects, and interaction terms between pre-1954 county-level characteristics and linear cohort trends. The income shock variable is proxied by the value of wealth assets. Controlling for the income shock effect is important considering the nonorthogonality between the diffusion of HYVs across regions and income shocks potentially impacting infant mortality. Furthermore, each regression also accounts for whether the childbirth involved assistance from a doctor, nurse, or midwife, as well as whether the pregnant mother underwent checkups by a doctor, aiming to mitigate the influences of delivery quality and prenatal health care on infant mortality. These interactions account for potential time-varying unobservables at the county level that could influence both the treatment variable and infant survival probabilities.

**Fig. 3.**
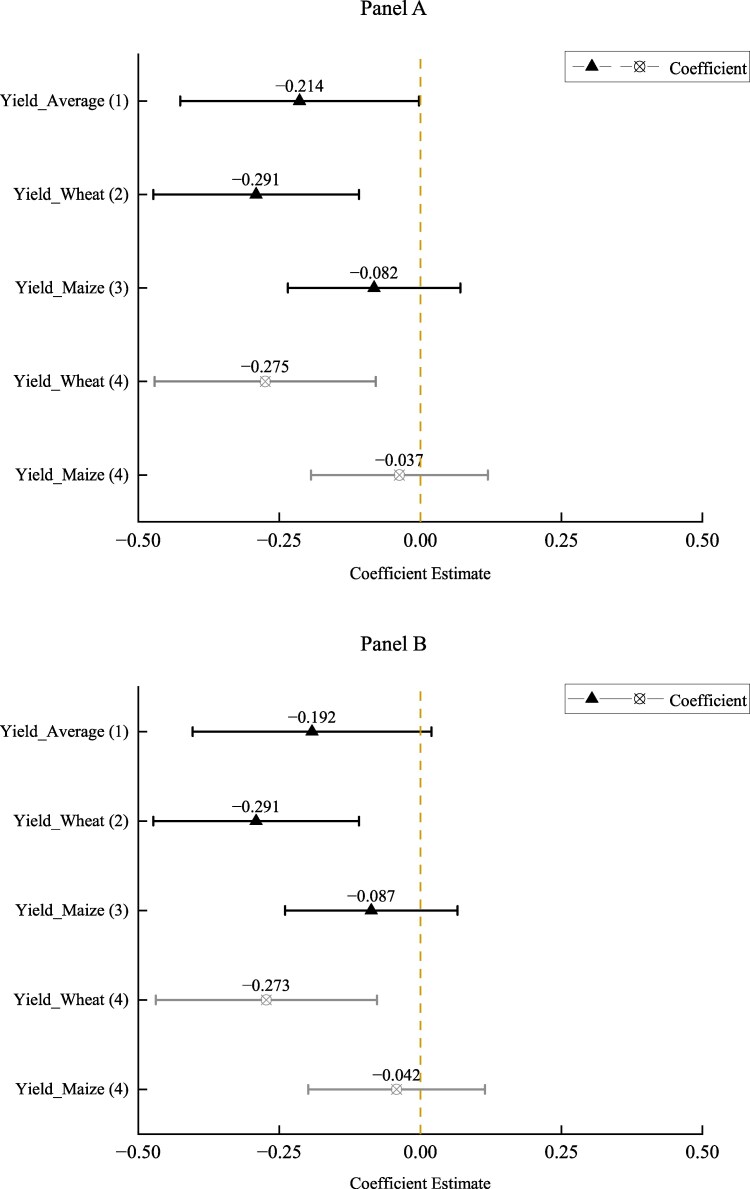
The main results of the effect of HYVs on infant mortality using rural sample. A) Yield average is the moving average of trial yield frontier of newly promoted HYVs of wheat and maize between lagged one and lagged three periods multiplied by the average suitability of wheat and maize. Yield wheat and yield maize represent moving average of average trial yield frontier of newly promoted HYVs between lagged one and lagged three periods of wheat and maize multiplied by their suitability, respectively. Yield_Average (1) represents the result of column (1) of Table S3. Yield_Wheat (2) represents column (2), Yield_Maize (3) represents column (3), and Yield_Wheat (4) and Yield_Maize (4) represent column (4) of Table S3, respectively. B) As a robustness check, we change the province-year fixed effects into the province-year time trend, and the results still hold. A) Baseline estimation. B) Robustness check.

Figure 3A displays the OLS results from estimating Eq. (1) and its variants. Here, we examined the trial yield frontier of combined wheat and maize HYVs, as well as that of wheat HYVs and maize HYVs separately, to assess their associations with infant mortality. Yield_Average (1) represents the result of column (1) of Table S3. Yield_Wheat (2) represents column (2), Yield_Maize (3) represents column (3), and Yield_Wheat (4) and Yield_Maize (4) represent column (4) of Table S3, respectively (detailed results are given in the Supplementary material Table S3).

The results indicated that the diffusion of HYVs was positively related to the reduction in infant mortality in rural China. Specifically, the increase in the average weighted yield frontier of wheat and maize from 1954 to 1987 (an accumulative increase of 0.903 log points) is associated with a reduction of infant mortality by ∼0.14 percentage points in regions with 1 SD larger of the average crop suitability (0.903 × 0.007 × 0.214). Furthermore, the negative and statistically significant coefficients of the proxy variables for prenatal health care suggest that the improvement of healthcare service is an important factor explaining a decrease in infant mortality over time.

Examining wheat and maize separately provides additional insights into the relationship between agricultural productivity improvement and infant mortality. Specifically, we find that the results are more significant and robust in the case of wheat as compared to maize, regardless of whether we define them as trial yield frontier or introduction of an additional newly released variety. The significant and negative coefficients of trial yield of wheat across all specifications offer robust evidence that the growth of agricultural productivity through the diffusion of wheat HYVs is positively correlated with infant's health outcomes. In contrast, while the coefficients of trial yield of maize are also negative, they lack statistical significance across all the specifications. These findings remain consistent when both the average weighted yield frontiers for wheat and maize were included in the same regression (see column 4 of Table S3). Once again, the coefficients for wheat are statistically significant, whereas those for maize are not. One possible explanation for these results is that traditionally, maize is not a major food for pregnant women (47). Additionally, the relatively lower promotion intensity and cultivated area of maize compared with wheat could also contribute to these findings. As indicated in Table 1, the average promoted number of maize HYVs was 1.504, significantly lower than that of wheat (4.098). Moreover, Fig. S4 illustrates that the average yearly cultivated area of maize by province was approximately half that of wheat in our sample.

Figure 3B presents the results from Eq. (2), where the province-specific time trend rather than province-year fixed effects was included. This specification accounts for possible unobserved trend variables that may nonlinearly vary by birth cohort in each province. All other control variables are the same as in Eq (1). The regression results are presented in the Appendix Table S4. The results are consistent with the baseline results presented in Fig. 3A. And the differentiated results between wheat and maize discussed above are also consistent here.

### Mechanism analysis

Several potential mechanisms may explain the pathways linking HYVs adoption and infants’ health outcomes. Although our dataset does not permit the examination of all possible mechanisms explaining the association between HYVs adoption and infant mortality, we focus on three specific pathways: increased grain output, changes in infant nutrition intake (e.g. breastfeeding), and shifts in maternal childbearing age.

First, we conducted a regression analysis to examine the relationship between HYVs diffusion and grain output, assessing the extent to which the diffusion of HYVs contributed to the growth of grain production. This analysis controlled for prefecture fixed effects, birth-year fixed effects, and province-year fixed effects. Our dataset comprises balanced panel data on grain output from 53 counties spanning the period from 1954 to 1966 in rural area. Figure S3 in the Supplementary material illustrates the trend of grain output over this period, showing a clear increase from an average of 82,678 mt in 1954 to 98,490 mt in 1966.

**Fig. 4.**
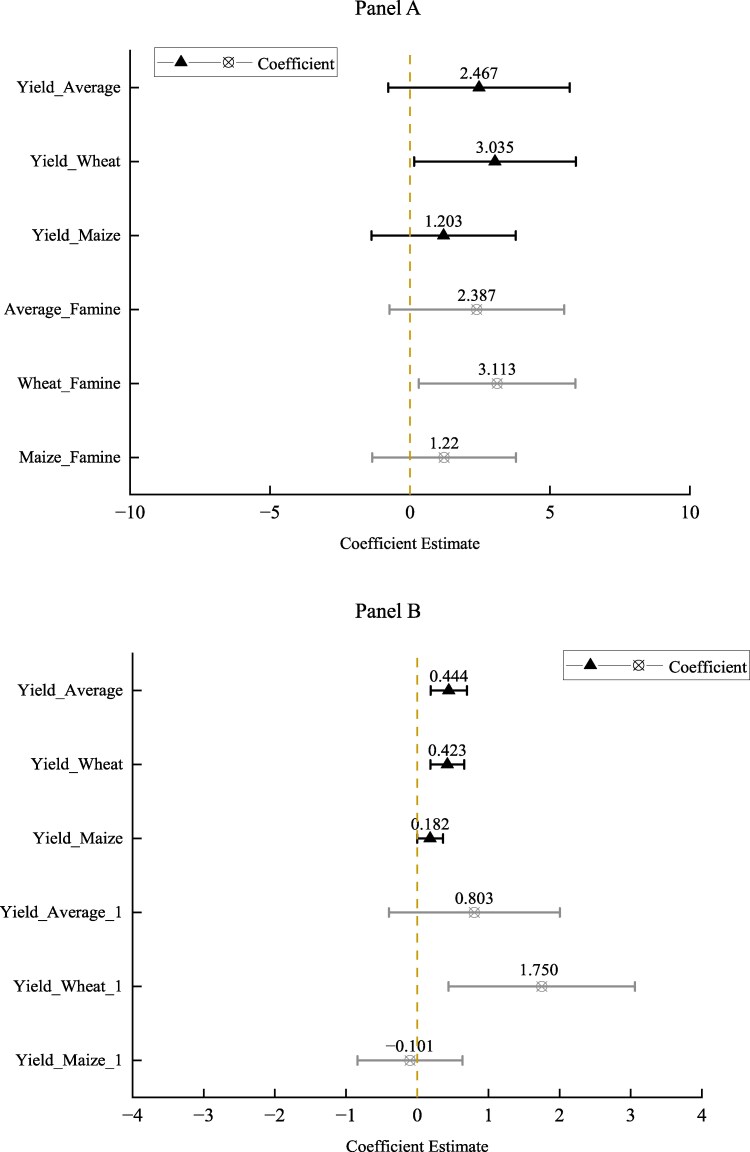
Mechanism analysis. A) The effects of HYVs on the grain output of 1954–1966. The line with the cross pointshadowed area represents the data which exclude the famine years. B) The effects of HYVs on infants’ breastfeeding. The line with the triangle point represents the effects of HYVs on whether infant is breastfeed. The line with the cross point represents the effects of HYVs on the month of breastfeed.

The estimation results of Fig. 4A indicate that most coefficients of interest were positive and statistically significant, affirming that the diffusion of HYVs is positively related to the increase in grain production. The point estimates are depicted by the triangle point. Previous studies have credited the growth of grain production for the remarkable reduction of infant mortality (48, 49). Therefore, grain production could serve as a mechanism for explaining the positive relationship between HYVs diffusion and reductions in infant mortality. While the diffusion of HYVs for both wheat and maize is positively correlated with their respective yields, the analysis suggests that, when considering the number of newly released varieties, the diffusion of maize HYVs has a stronger positive relation with grain yield compared with wheat. However, it is important to note that wheat provides greater nutritional benefits for pregnant women, making it a primary factor in directly reducing infant mortality. In contrast, the diffusion of maize HYVs may indirectly influence infant mortality rates by enhancing income and through various other socioeconomic mechanisms.

To further investigate the relationship between HYVs diffusion and grain production, we conducted a robustness check by excluding the famine years (1959–1961), during which grain production sharply declined to an average level of around 60,000 mt (Fig. S3). This exclusion is warranted as previous studies have identified the decrease in grain production as a key contributor to the Great Famine (50, 51). The point estimates of this set of regressions are represented by the line with the cross point (detailed results are given in the Supplementary material Table S5), showing that the coefficients of interest remain statistically significant across most specifications. These findings suggest that the positive relationship between HYVs diffusion and grain production is robust, regardless of whether the famine years are included or not. Moreover, the diffusion of HYVs appears to have mitigated the effects of the Great Famine to some extent. In other words, the severity of the famine in 1959–1961 could have been even greater without the increase in grain production facilitated by HYVs diffusion.

Second, we analyzed the relationship between HYVs adoption and breastfeeding practices, which directly influence infants’ heath—particularly during the period before 1980s when milk powder was scarce in rural China. As breastmilk was the primary source of nourishment for infants, breastfeeding provided protective benefits and reduced the risk of infant mortality (52). Following the works of Singh and Kumar (53) and Sayed and Schönfeldt (54), we used indicators such as whether an infant was breastfed and the duration of breastfeeding, to examine the relationship between HYVs adoption and breastfeeding practices. These practices, in turn, have direct implications for nutritional intake of newborns. The results of this analysis are reported in Fig. 4B.

The analysis reveals a strong connection between HYVs adoption and breastfeeding practices, as evidenced by the positive and significant coefficient of $\mathrm{Suitabilit}y_{c}\times \mathrm{HY}V_{s,y-3∼y-1}$ for almost all the regressions (the point estimates are depicted by the line with the triangle point). A straightforward calculation reveals that the accumulative gain in average weighted yield frontier of wheat and maize HYVs from 1954 to 1987 (amounting to an accumulative increase of 0.903 log points) resulted in an approximate 0.28-percentage-point rise in the likelihood of an average infant being breastfed in regions with 1 SD higher than the average crop suitability (0.903 × 0.007 × 0.444). Similarly, as a robustness check, the introduction of an additional newly promoted variety for wheat or maize is associated with an approximate 0.22-percentage-point increase in the probability of an average infant being breastfed in areas with 1 SD greater than the average crop suitability (1 × 0.007 × 0.314) (detailed results are given in the Supplementary material Table S6).

Apart from the increase in the likelihood of being breastfed on the extensive margin, the analysis also shows that HYVs were positively related to the number of months for which an infant was breastfed on the intensive margin (see the line with the cross point of Fig. 4B). When the yield frontiers (or the numbers of newly promoted varieties) of wheat and maize are both included in the same regression equation, the results indicate that wheat yield or varietal improvement is more strongly associated with the increase in breastfeeding practices than maize, in terms of both the extensive and intensive margins. Notably, the results presented in Fig. 4B exhibit a nearly mirror pattern compared with those of the main infant mortality regression results (Fig. 3) in terms of sign and significance level. Therefore, breastfeeding practices appear to be an important mechanism underlying the positive relationship between HYVs diffusion and reductions in infant mortality.

Third, we examined the relationship between HYVs diffusion and maternal childbearing age. The demographic profile of mothers may be closely linked to income changes resulting from HYVs adoption. For example, risk-averse mothers may delay fertility decisions when income is insufficient, while those with fewer financial constraints are more likely to conceive at an optimal time, potentially reducing infant mortality rates (55). To explore whether maternal selection in fertility decisions could be a mechanism, we regress mother's age at the time of childbirth on the diffusion of HYVs. The regression results are presented in Table S7. The coefficients indicate whether HYVs diffusion influenced childbearing decisions. Our approach was inspired by the methodology of Bharadwaj et al. (12). However, the results showed no statistically significant coefficients, suggesting that HYVs diffusion did not have a substantial influence on childbearing decisions.

## Robustness checks

We also carried out several robustness checks to further corroborate our baseline results. The first robustness check examined whether the baseline results are influenced by the definition of HYVs. In the baseline analysis, we defined HYVs as the moving average of 3-year lag to 1-year lag, assuming that gestation typically lasts 9 or 10 months, and the diffusion of HYVs could lag accordingly. To test the robustness of this baseline definition, we experimented with different moving averages, ranging from a 2-year lag or 5-year lag to a 1-year lag. The results of this check (reported in Tables S8 and S9) are highly consistent with those reported in Fig. 3, suggesting that the positive and significant relationship between HYVs adoption and reductions in infant mortality are not driven by the specific construction of the HYVs variable.

The second robustness check assessed the sensitivity of the baseline results to the inclusion of early neonatal deaths. Table S10 reports the results after excluding infants who died within a week of birth (i.e. early neonatal deaths). This adjustment helps eliminate the potential confounding effects from birth defects and neonatal asphyxia—common causes of neonatal deaths that are not directly related to the diffusion of HYVs. The results were largely consistent with our benchmark estimates, though the estimates were slightly lower than the baseline.

The third robustness check examines whether our baseline results are influenced by the inclusion of the famine years. The influences of the Great Famine on infant mortality may vary across counties, potentially altering the relationship between HYVs adoption and infant mortality (56, 57). There is ongoing debate over the fundamental aspects of the Great Famine. Even though there is lack of robust evidence, the Great Famine's death toll exceeds that of any other recorded famine, based on crude death numbers (58) (see Fig. S2 and Table S2 of the Supplementary material).^m^ Another key issue is the cause of the Great Famine.^n^ It was closely associated with a reduction in grain production, resulting from disruption in production caused by the Great Leap Forward campaign and the collectivization of agriculture (59), which led to a drastic fall in grain production from 1959 to 1961 (57).

However, it is generally accepted that the decline in food availability alone could not account for the estimated 20 to 30 million excess deaths between 1958 and 1961 (50). Note that the statistics from the HYVs dataset showed that the adoption of HYVs continued both before and during the Great Famine. We were concerned that including the famine years in our study might lead to an overestimate of the relationship between the adoption of HYVs and infant mortality. The key feature of the Great Famine included a substantial reduction in fertility rate and increased mortality rates across all age cohorts. The diffusion of HYVs may have had a larger effect during the famine time, potentially driving our estimates upward. Therefore, in our study, we expanded on the existing literature by examining whether the diffusion of HYVs, which is expected to increase agricultural production, was associated with reductions in infant mortality during the ordinary times, aside from the specific famine years.

To test the robustness of our estimates, we reestimated the model by excluding the famine years in our sample (see Table S11). The results were largely consistent with our baseline estimates, suggesting that our findings were not driven by the famine year period. Additionally, we explored the spatial relationship between areas most affected by the Great Famine and those that benefited from the HYVs adoption, which could complicate our results. Table S12 shows no correlation between the severity of the famine and productivity in the pre- or postfamine years at the county level using the average HYVs adoption values 3 years before or after the famine as a robustness check. The results further support and consolidate our main results.

Fourth, we test the sensitivity of our results to the exclusion of Guizhou and Shanghai where rice production is significant. Table S13 presents the results, which are highly consistent with our baseline results. The geographic distribution of China's in-depth fertility data is shown in Fig. S1.^o^

Fifth, while our baseline results are based on the rural subsample, we present the results based on the combined sample of rural and urban *hukou* groups in Appendix Tables S15 and S17 for reference. The descriptive statistics of the combined sample are presented in Table S14. The results are highly consistent with the baseline results using only the rural sample, suggesting that improvements in HYVs also indirectly benefited urban residents.

Finally, to address the underrepresentation of older women in earlier years of the recall period, we have constructed sampling weights based on age-group populations. Following the method used by the China Family Panel Studies (60), we calculated weights by comparing the number of women in different age groups (*N*s) in our sample regions for each year with corresponding age-group population (*N*c) from the 1953, 1964, and 1982 censuses. Specifically, the weight for each age group is the reciprocal of the ratio of the population from the sample (*N*s) and the population from the census (*N*c) for the closest year, that is, that the weight is *N*c/*N*s. The estimated results with sample weights in Tables S16 and S18 are qualitatively consistent with the unweighted findings (see Tables S15 and S17).

## Heterogeneity analysis

**Fig. 5.**
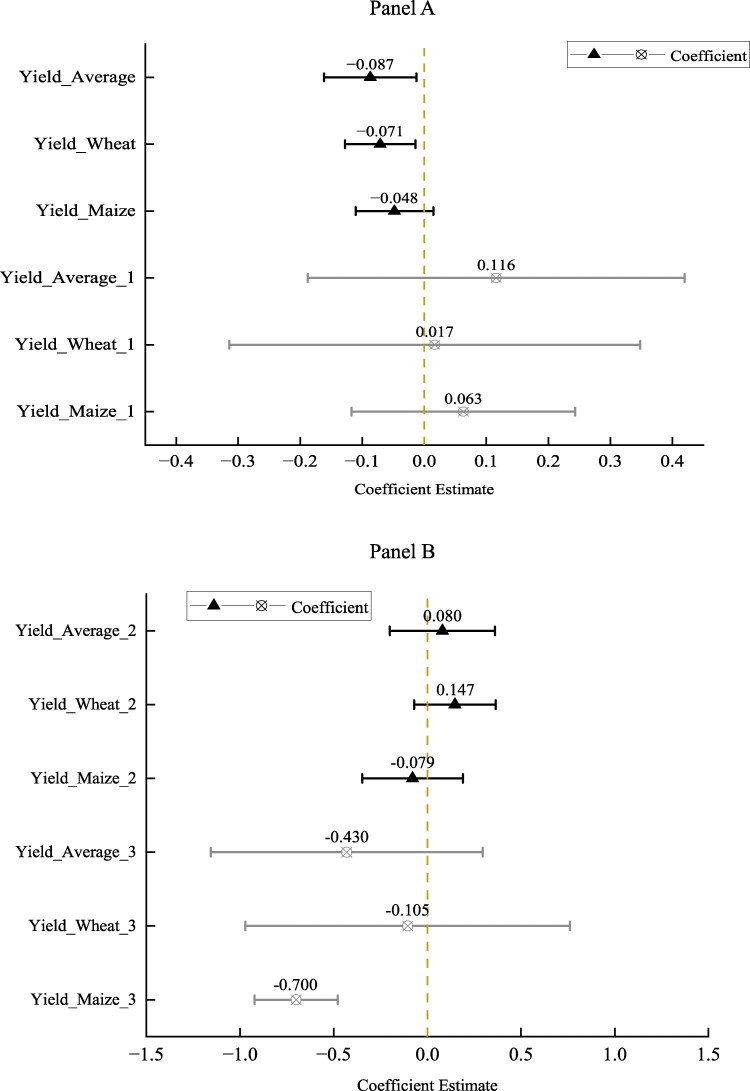
Heterogeneous effects. A) The heterogeneous effects of HYVs on the infant mortality. The line with the triangle point represents the heterogeneous effects of infant gender. The line with the cross point represents the heterogeneous effects of birth control. B) The heterogeneous effects of HYVs on the infant mortality. The line with the triangle point represents the heterogeneous effects of mother age. The line with the cross point represents the heterogeneous effects of mother education.

In this study, we further explored the heterogeneous relationships between HYVs adoption and infant mortality across various factors, including the infant's gender, the period before or after the introduction of birth control measures, mother's age at the infant's birth, and the mother's educational attainment. To accomplish this, we included additional interaction terms between these variables and HYVs diffusion variables. The results of this heterogeneity analysis are reported in Fig. 5.

The line with the triangle point in Fig. 5A indicates the heterogeneous relationships across the infant's gender. The results indicated that the positive relationship between HYVs adoption and reductions in infant mortality was more pronounced on male infants than female infants. This finding could be attributed to the culture preferences for son among Chinese parents, rooted in the Confucian ideology (61, 62). Additionally, male fetuses are shown to be more vulnerable than the female fetuses (63, 64). Therefore, it is plausible to conclude that nutritional improvements resulting from HYVs adoption had a greater health benefits for male infants than female infants.

The line with the cross point in Fig. 5A illustrates the heterogeneous relationship before and after the implementation of China's birth control policy, which began in 1982. This family planning policy was a government measure aimed at regulating and guiding population behaviors related to marriage and childbirth, with the goal of aligning such behaviors with planned demographic objectives.^p^ The insignificant coefficients of the interaction term between the birth control policy dummy and HYVs adoption across all specifications tend to suggest that the association between HYVs adoption and infant mortality remained robust before and after the implement of birth control policy. In other words, the introduction of birth control measures did not significantly alter the relationship between HYVs adoption and infant mortality rates.

The line with the triangle point in Fig. 5B shows the heterogeneous correlations between HYVs adoption and the infant mortality by mother's age at the infant's birth year. Specifically, we defined an age dummy variable that equals to one if the mother was 35 years old and above, and zero otherwise (65). The coefficients of the interaction terms between the age dummy and HYVs adoption are statistically insignificant across all specifications. These findings suggest that the positive relationship between HYVs adoption and the reduction in infant mortality is unlikely to be influenced by mother's age at birth. In other words, the introduction of HYVs benefited mothers of all pregnancy ages.

The line with the cross point in Fig. 5B demonstrates the heterogeneous relationships between HYVs adoption and infant mortality across mother's education attainment. We hypothesize that mother's education may be correlated with the relationship between HYVs adoption and infant mortality. Specifically, we expect the less educated mothers to potentially benefit more from the diffusion and adoption of HYVs (12). To analyze this, we defined an education dummy variable that equals to one if the mother had completed high school, and zero otherwise (66). The highly insignificant coefficient of the interaction term between the education dummy and HYVs adoption across all specifications suggests that the relationship between HYVs adoption and infant mortality was independent of the mother's education attainment. Detailed results are also given in Table S19.

Additionally, we also considered the total number of wheat and maize varieties together, as well as their individual numbers to account for the diffusion of HYVs. The results remained largely consistent with our baseline results. For detailed results, refer to the Supplementary material Table S20.

## Conclusion

While the contribution of high-yielding crop varieties (HYVs) to global aggregate food supply is widely recognized, there has been a lack of microlevel evidence on the relationship between HYVs adoption and human welfare, including health outcomes. The primary contribution of our study is to address the limited evidence on the relationship between agricultural productivity growth and infant mortality. We analyzed the diffusion of HYVs of wheat and maize (two major staple crops in China) and its association with infant mortality using data from 1954 to 1987. The analysis revealed that HYVs, together with improvement in healthcare services, had a substantial positive association with reductions in infant mortality during the study period. This main finding remained largely unchanged even when the Great Famine period was excluded from the sample. This finding is also robust to different definitions of HYVs and the control of health improvement and income mechanism. We also identified the heterogeneous relationships between HYVs adoption and reduction in infant mortality across a range of factors, including infant gender, mother's characteristics, and geographic areas.

This study contributes to the evidence supporting the beneficial effect of adoption and diffusion of HYVs on human welfare, particularly in reducing infant mortality. The findings of this study hold significant policy implications for the low- and middle-income countries grappling with food and nutritional insecurity. In these nations, strengthening governmental and public efforts to develop new agricultural technologies, including HYVs, and promoting their widespread adoption emerges as a recommended strategy for achieving food security and reducing infant mortality.

In conclusion, it is important to note a caveat regarding our study's findings. The exclusion of women who perished due to childbirth or famine-related causes, along with the associated infant deaths, may result in an underestimation of the overall infant mortality rates. Although we are unable to directly address this limitation due to the constraints imposed by our dataset, we believe that our parameter estimates provide a conservative lower bound for these rates. Future research should aim to incorporate complementary data sources or advanced modeling techniques to bridge this gap and offer a more comprehensive understanding of infant mortality patterns.

## Supplementary Material

pgaf048_Supplementary_Data

## Data Availability

The data supporting the findings of this study can be accessed at Science Data Bank: http://doi.org/10.57760/sciencedb.25363, including the infant mortality dataset (comprising characteristics of the infant, the infants' mother and father, etc.), HYVs dataset (comprising the trial yield of wheat and maize varieties, number of wheat and maize cultivars, etc.). All empirical analyses were performed with Stata 17, and code for all figures and tables can be accessed at Science Data Bank: http://doi.org/10.57760/sciencedb.25363

## Footnotes

a The information can be accessed through: https://www.who.int/news-room/fact-sheets/detail/newborns-reducing-mortality.

b All the provinces and municipalities included in our sample, with the exception of Guizhou and Shanghai, are situated in the northern Huang-Huai-Hai area, known for its significant production of wheat and maize. To ensure the reliability of our findings, we also conduct the analysis excluding the samples from Guizhou and Shanghai, which is in alignment with our initial regression analysis. Ideally, we would incorporate rice data for a comprehensive robustness check; however, we do not possess data on enhanced rice varieties for the same time.

c The information can be accessed through: https://data.worldbank.org/indicator/SP.DYN.IMRT.IN.

d The information can be accessed through: https://www.nature.com/articles/517247d.

e Due to technical problems, the data collected in Guangdong Province had too many missing variables and were excluded from this study.

f Both rounds of the China In-depth Fertility Sample Survey (1985 and 1987) employed rigorous sampling methodologies, including stratified, staged, probability-proportional-to-size, and random equidistant sampling techniques. The final stage sampling ensured representativeness by selecting households proportionally and identifying all eligible women. The sampling procedure is reported in Appendix S1.2 of the Supplementary material. The details of these procedures demonstrate the robustness of the sampling strategy across provinces. While minor variations in methods existed between regions (Hebei, Shaanxi, and Shanghai), the survey completion rates were consistently high (above 97%), further supporting the reliability of the data.

g Results based on both rural and urban *hukou* are also included in the Appendix of Table S15 for the reference.

h In China, the subnational jurisdictions consist of four layers of government: the provincial level, the prefectural level, the county level, and the township level.

i Following Nunn and Qian (13) and Chen and Kung (17), the suitability of cultivation defined by soil, slope, and climatic characteristics better explains the land suitability of certain staple crops. The suitability of cultivating maize and wheat was calculated at county c, province s from the grid data (Food and Agriculture Organization 2002, Global Agro-Ecological Zones [GAEZ] database). The data measured the suitability for cultivating individual crops at a very disaggregated geographic level. When we used the moving average of total number of newly promoted high-yielding varieties or trial average yield frontier of newly promoted varieties in province s, the average suitability of maize and wheat was calculated by:

$$\mathrm{Suitabilit}y_{\mathrm{maize}}\times \frac{\mathrm{Sown}\;\mathrm{area}\;\mathrm{of}\;\mathrm{maize}}{\mathrm{Sown}\;\mathrm{area}\;\mathrm{of}\;\mathrm{maize}+\mathrm{Sown}\;\mathrm{area}\;\mathrm{of}\;\mathrm{wheat}}+\mathrm{Suitabilit}y_{\mathrm{wheat}}\times \frac{\mathrm{Sown}\;\mathrm{area}\;\mathrm{of}\;\mathrm{wheat}}{\mathrm{Sown}\;\mathrm{area}\;\mathrm{of}\;\mathrm{maize}+\mathrm{Sown}\;\mathrm{area}\;\mathrm{of}\;\mathrm{wheat}}.$$

j Dummy variables record households’ possessions of the following items: bicycles, clocks, radios, televisions, sewing machines, washing machines, and refrigerators.

k The weather data were obtained from the China Meteorological Data Sharing Service System (CMDSSS), which records daily minimum, maximum, and average temperatures, and precipitation for 820 weather stations in China. CMDSSS was developed and is currently managed by the Climatic Data Center, National Meteorological Information Center, China Meteorological Administration. See https://data.cma.cn/en for details. The dataset contains the exact coordinates of each weather station, enabling us to merge the weather data with our agricultural data. For counties with several weather stations, we constructed weather variables by taking the simple average of the weather variables across these stations. For counties without a weather station, we imputed the weather information from the neighboring counties for these counties.

l We recognize that the exclusion of women who died due to childbirth or famine-related factors, as well as their associated infant death, could lead to an underestimation of overall infant mortality. While we cannot directly resolve this limitation due to the constraints of the dataset, we consider our parameter estimates to represent a lower bound of the infant mortality rates.

m We can see from Fig. S2 the geographic distribution of the infant mortality rate in the famine year.

n Other questions about the Great Famine could be “why it ended in 1962?”, “Why it was more severe in the grain production province?”, etc.

o From the consumption side of the staple crops, rural household in China heavily rely on their own production, even till the mid of 1980s. This justifies us only use the wheat and maize as the shock of agricultural productivity. Ideally, we should include rice data to conduct the robustness test; it is pity that currently we do not have the rice varieties.

p More details about the birth control policy could be found in Ebenstein (67) and Li *et al.* (68).
