# Correction to: The influence of improved wheat and maize varieties on infant mortality in China

**DOI:** 10.1093/pnasnexus/pgaf224

**Published:** 2025-07-25

This is a **correction** to:

Xiaobing Wang, Xinyu Liu, Shi Min, Songqing Jin, Jikun Huang, Scott Rozelle, Jieyuan Feng, Boddupalli M Prasanna, The influence of improved wheat and maize varieties on infant mortality in China, *PNAS Nexus*, Volume 4, Issue 2, February 2025, pgaf048, https://doi.org/10.1093/pnasnexus/pgaf048

The authors have provided an updated data availability statement for this paper.

