# Supplementary material for: The influence of improved wheat and maize varieties on infant mortality in China
Source: PNAS Nexus. 2025 Feb 20;4(2):pgaf048. doi: 10.1093/pnasnexus/pgaf048 (PMC11840862; doi:10.1093/pnasnexus/pgaf048)
Supplement: pgaf048_Supplementary_Data [file pgaf048_supplementary_data.docx]

**Supporting information**

**S1. The details about the infant mortality dataset**

**1.1 The contents of the infant mortality dataset**

The fertility surveys, which were nationally representative surveys of women of child-bearing age, had three sections:

First, an individual questionnaire collected the characteristics of all eligible women -- specifically, married women aged above 14 and below 50 years old during the survey year. In total, the survey sampled more than forty thousand eligible women and collected data on aspects such as a woman’s birth year, educational attainment, rural or urban household registration (hukou), and marital history.

Second, the fertility surveys collected the complete birth histories of the eligible women; on average, each woman had birthed 3.6 infants. This section chronicled births as far back as 1954, and sampled more than ten thousand infants born between 1954 and 1987. It collected data on pregnancy details, birth histories, prenatal care, delivery circumstances, and neonatal healthcare aspects such as infant vaccinations. In this study, to quantify infant mortality and select variables for the heterogeneity analyses, we use the infant’s year of birth, their birth order, an indicator marking a multiple birth (e.g. twins), a dummy for the female sex, and the length of the infant’s life.

Third, fertility surveys collected information about the households of the sampled women, including the condition of their housing and their possession of durable assets.

The coherence between the fertility survey-based infant mortality trend and official statistics implies the survey’s representation of women. Comparability can be drawn between the questionnaire of the fertility surveys and those of Demographic and Health Surveys (DHS) conducted in other developing nations.

We could see from Figure 1 that there is a declining trend of infant mortality in China, we then present a more detailed analysis of the pattern during the Great Famine. The infant mortality rate experienced a peak from 1959 to 1961, coinciding with the Great Famine. Before this period, infant mortality in China had been on a downward trajectory. The initiation of the Great Leap Forward in 1958 resulted in the establishment of extensive people's communes and a rationing system for food distribution (1-2). Widespread famine ensued when food supplies became insufficient, affecting both urban and rural areas (3-4).

During the 1959 to 1961 Great Famine, two distinct patterns emerged: a sharp increase in the mortality rate and a notable decrease in the fertility rate (2,5). Despite ongoing debates about the extent of population loss and infant mortality rate during the Great Famine, demographers have provided varying estimates, ranging from approximately 15 to 45 million individuals, based on census and demographic data (5-7). Despite this lack of consensus, it is evident that, even though the Great Famine was short-lived, the fertility rate during those years plummeted from 34 to 21 per 1000 (2).

Our infant mortality dataset recorded 2860 births and 367 infant deaths during 1959 to 1961. This data aligns with the statistics calculated using the National Bureau of Statistics, revealing an average infant mortality rate of 128.3‰ during the Great Famine, peaking in the early 1960s and 35.5‰ higher than just before the onset of the Great Famine in 1958. This indicates that the Great Famine constituted a nutrition and health shock for both infants and children and had lasting effects on health outcomes (8).

**1.2 The sampling procedure of the datasets**

*1.2.1 Sampling procedure of the 1985 China In-depth Fertility Sample Survey*

This survey used stratified, staged, probability proportional, and random equidistant sampling methods. The final stage of sampling adopts the equal probability method to select households, and then all qualified women are identified from the households. In terms of specific methods, there are slight differences between Hebei, Shaanxi, and Shanghai.

a) In Hebei and Shaanxi provinces, the basic sampling units are cities and counties. These are divided into three levels based on geography: cities, counties, and mountainous counties. The number of units at each level is determined based on the proportion of the level's size to the total size of the province. Using probability proportional to size (PPS), 34 basic sampling units from Hebei and 29 from Shaanxi are selected in geographical order. Three major cities with large populations are considered self-representing layers, with a probability of 1. After selecting the basic sampling units, regional sampling is carried out in the final three stages, all selected using a probability system proportional to the estimated population size based on the list. Four secondary sampling units (urban streets or rural townships and towns) are selected from each basic sampling unit. From the selected secondary units, two third-level sampling units (residents' committees in urban areas and villagers' committees in rural areas) are chosen. Finally, two regional units are selected from each third-level sampling unit, namely the resident group in urban areas and the village group in rural areas.

b) In Shanghai, the selection process is divided into three stages. The first stage of county-level selection is omitted. The basic sampling units are at the street, town, or township level, which are treated as the corresponding three levels. A probability system proportional to population size is used to select 54 basic sampling units. From each selected basic sampling unit, 3 neighborhood (or village) committees are chosen, and then 3 neighborhood (or village) groups are selected from each of these committees.

*1.2.2 1987 Second China In-depth Fertility Sample Survey*

According to the sample design, 7,000 married women under the age of 50 were selected in Beijing, and 6,000 women were selected in each of the other five provinces to represent the fertility level of their respective provinces and cities. The sampling probabilities for the five provinces and one city are approximately 3.6‰ for Beijing, 0.92‰ for Liaoning, 0.44‰ for Shandong, 0.68‰ for Guangdong, 1.43‰ for Guizhou, and 1.64‰ for Gansu. The sampling method follows the principles of stratification, staged sampling, probability proportional to size, and random equidistant sampling. A total of 858 townships, towns, and streets from 210 counties and cities (districts), as well as 2,194 village committees and neighborhood committees, were selected in the five provinces and one city. In total, 49,458 households were surveyed, with a completion rate of 97.7%; 39,210 married women of childbearing age completed a personal survey, with a completion rate of 98.5%. Of these, 7,622 women from 9,929 households were surveyed in Beijing, with a completion rate of 98.6%; Liaoning Province surveyed 6,567 women from 8,459 households, with a completion rate of 96%; Shandong Province surveyed 6,124 women from 8,238 households, with a completion rate of 99.6%; Guangdong Province surveyed 6,654 women from 8,266 households, with a completion rate of 98.2%; Guizhou Province surveyed 6,489 women from 7,957 households, with a completion rate of 99.3%; Gansu Province surveyed 5,754 women from 6,609 households, with a completion rate of 97.1%. Due to factors such as household relocation and illness among the surveyed women, 1,170 households and 573 women could not be surveyed.

| 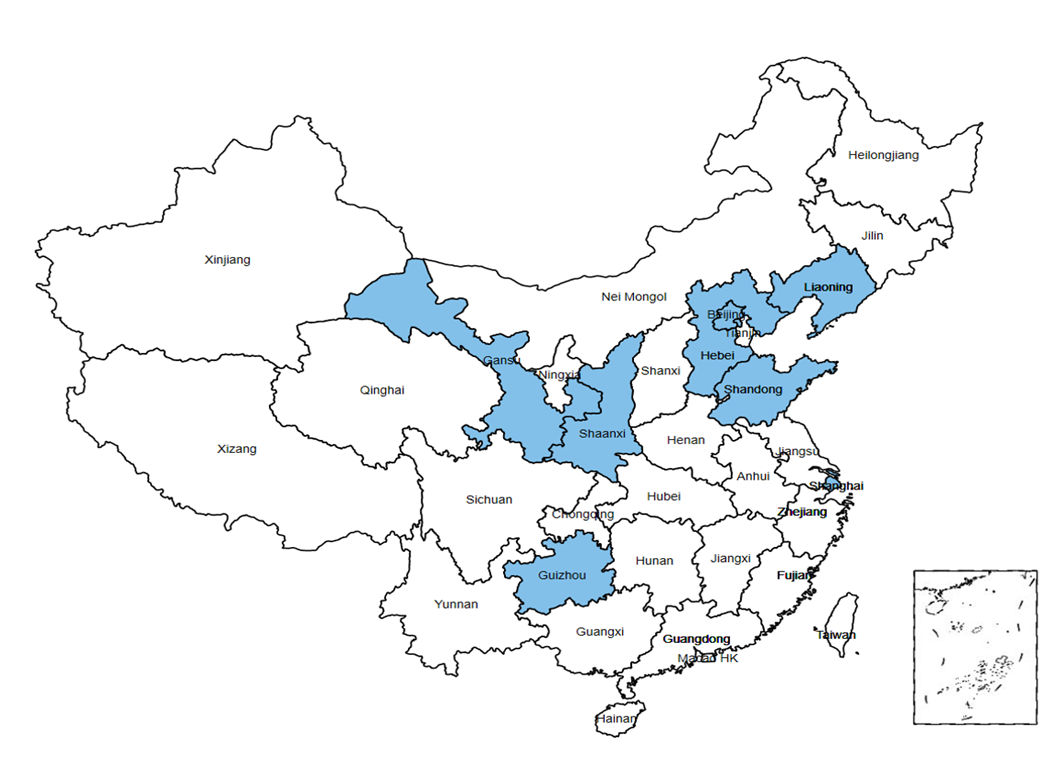  The national distribution of the sample | | | |
| --- | --- | --- | --- |
| 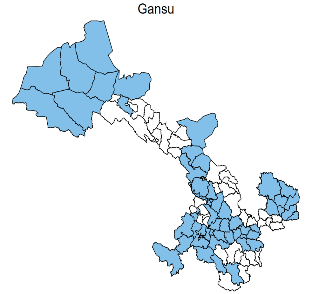 | 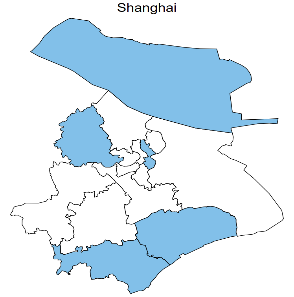 | 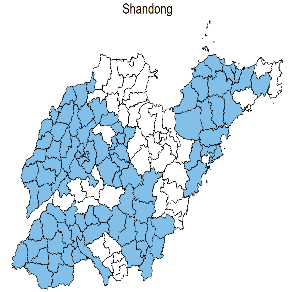 | 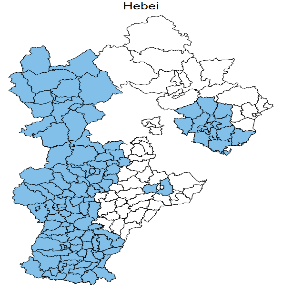 |
| (1) Gansu | (2) Shanghai | (3) Shandong | (4) Hebei |
| 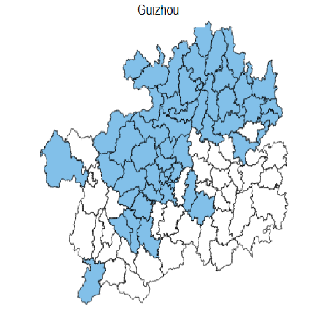 | 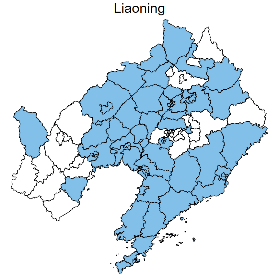 | 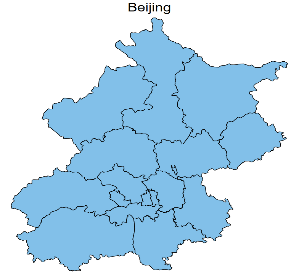 | 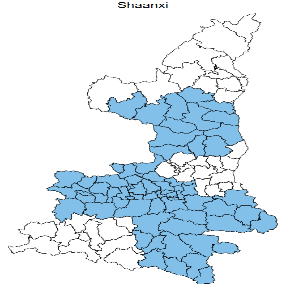 |
| (5) Guizhou | (6) Liaoning | (7) Beijing | (8) Shaanxi |
| The county level distribution of the sample | | | |

Figure S1. The geographic distribution of our sample


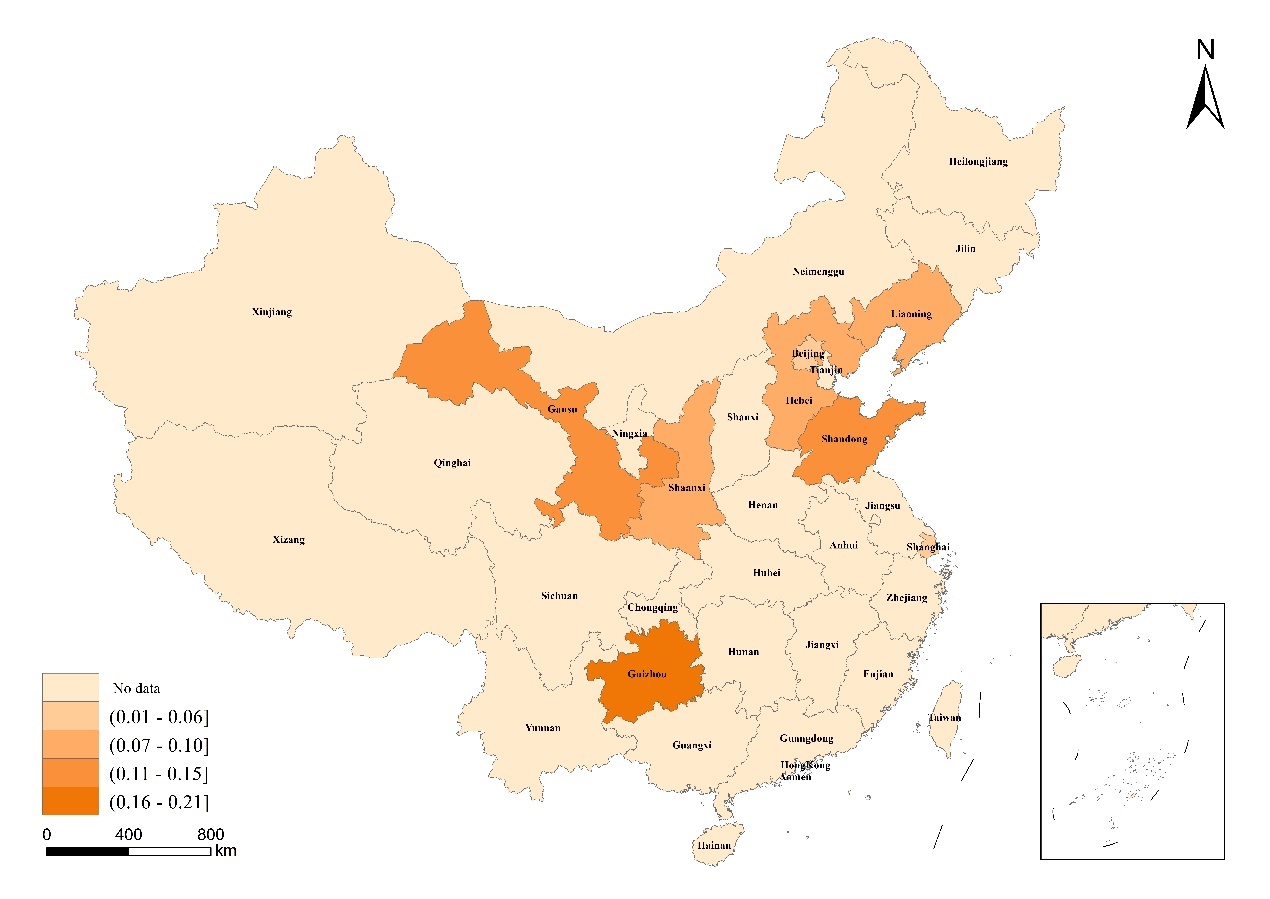


Figure S2.The average infant mortality rate during the 1959-1961 famine

Figure S3. The trend of grain output over 1954-1966.

Notes: The data source is from compilation of population statistics and local gazettes.

Figure S4. The sown area of maize and wheat over 1954-1986.

Notes: The data source is from the National Bureau of statistics.

Table S1. Variable definition

| Variable Definition | Description |
| --- | --- |
| ***Dependent variable*** |  |
| Whether infant died | Whether the child dies twelve months after birth |
| ***Independent variable*** |  |
| Lnyield_wheat | Average test yield frontier of newly promoted bred/introduced varieties (by province of application and promotion) for Wheat (log(Kg/mu)) |
| Lnyield_maize | Average test yield frontier of newly promoted bred/introduced varieties (by province of application and promotion) for Maize (log(Kg/mu)) |
| Lnyield_aver | Lnyield_wheat multiplied by weighted sown area of wheat plus the Lnyield_maize multiplied by weighted sown area of maize |
| Num_wheat | Number of newly promoted bred/introduced varieties (by province of application and promotion) for Wheat |
| Num_maize | Number of newly promoted bred/introduced varieties (by province of application and promotion) for Maize |
| Amount | Number of newly promoted bred/introduced varieties (by province of application and promotion) for Wheat and Maize |
| Suit_wheat | Crop suitability of Wheat/100 |
| Suit_maize | Crop suitability of Maize/100 |
| Suit_aver | Suit_wheat multiplied by weighted sown area of wheat plus the Suit_maize multiplied by weighted sown area of maize |
| ***Control variable*** |  |
| *Individual level* |  |
| Child Male | Whether the child is male |
| Birth Order Number | Birth order of the child |
| Child Multiple | Whether the child was part of a twin birth |
| Boy before | Whether the family has already got a boy before the child is born |
| Birth weight | Weight of the child (500g) |
| Mother edu | Whether the respondent mother has received senior high or more education (1=yes; 0=otherwise) |
| Mother work | Whether the respondent mother has worked before |
| Mother age at child birth | Age of the respondent mother when she gave birth to the baby |
| Father age at child birth | Age of the respondent father when he had a baby |
| Wealth_index | Asset of the household |
| Urban | Whether the household to which the respondent mother belongs is in an urban area |
| Place_assist | Whether the child delivered at home, hospital or clinic is assisted by doctor, nurse or midwife(1=yes; 0=otherwise) |
| Pregnant_check | Whether the respondent mother has pregnant check by doctor in the first 6 months (1=yes; 0=otherwise) |
| *County level* |  |
| Grain production | Grain production (Tons) |
| Teacher number | The number of teachers |
| School number | The number of schools |
| GDP | Gross regional product (10000 yuan)/registered residence population each year (person) |
| Industry ratio | Value added of the secondary industry (RMB 10000)/Gross Domestic Product (RMB 10000) |
| Service ratio | Value added of the third industry (RMB 10000)/Gross Domestic Product (RMB 10000) |
| Family savings | Savings deposit balance of urban and rural residents (10000 yuan)/registered residence population (person) |
| Fixed asset | Fixed assets investment (10000 yuan)/registered residence population (person) |
| Government expenditure | Public fiscal expenditure (10000 yuan)/regional GDP (10000 yuan) |
| Population density | Registered residence population (10000 persons)/administrative area (km2) |
| Average temperature | Average yearly temperature at the county level (degrees Celsius) |
| Average precipitation | Average yearly rainfall at the county level (millimeters) |
| *Province level* |  |
| Cultivated area | Cultivated area per capita at the province level (1000 Ha) |
| Quota | The amount of grain the central government buys from local governments at the province level (10000 tons) |
| Ln_gdp | ln (gross regional production per capita) at the province level (yuan) |

Notes: The data sources from which the variables come from are the 1985 First China In-depth Fertility Sample Survey and 1987 Second China In-depth Fertility Sample Survey. The county level statistics are hand collected by ourselves from *Wanfang Local Chronicles Knowledge Service Platform* and *China Economic and Social Data Research Platform*.

Table S2. Death rates (‰) and excess death rates (‰) in the sample provinces

| Provinces | Death rates (‰) | | | |  | Excess death rates (‰) | | |
| --- | --- | --- | --- | --- | --- | --- | --- | --- |
|  | 1956-1958 | 1959 | 1960 | 1961 |  | 1959 | 1960 | 1961 |
| Beijing | 8.00 | 9.70 | 9.10 | 10.80 |  | 1.70 | 1.10 | 2.80 |
| Hebei | 11.17 | 12.30 | 15.80 | 13.60 |  | 1.13 | 4.63 | 2.43 |
| Liaoning | 8.27 | 11.80 | 11.50 | 17.50 |  | 3.53 | 3.23 | 9.23 |
| Shanghai | 6.30 | 7.80 | 6.90 | 7.70 |  | 1.50 | 0.60 | 1.40 |
| Shandong | 12.33 | 18.20 | 23.60 | 18.40 |  | 5.87 | 11.27 | 6.07 |
| Guizhou | 13.57 | 20.30 | 52.30 | 23.30 |  | 6.73 | 38.73 | 9.73 |
| Shannxi | 10.40 | 12.70 | 12.30 | 8.80 |  | 2.30 | 1.90 | -1.60 |
| Gansu | 14.40 | 17.40 | 41.30 | 11.50 |  | 3.00 | 26.90 | -2.90 |

Source: China Compendium of Statistics 1949-2008 (National Bureau of Statistics of China, 2010).

Note: Excess death rates are calculated as the difference between death rates in the famine year and the average of the death rates in 1956-1958

Table S3. The coefficients of HYVs on the infant mortality from 1957-1987 using rural sample

|  | Whether infant died (1=yes, 0=no) | | | | | | | |
| --- | --- | --- | --- | --- | --- | --- | --- | --- |
|  | (1) | (2) | (3) | (4) | (5) | (6) | (7) | (8) |
| Lnyield_aver (t-3~t-1)***suit**aver | -0.214** |  |  |  |  |  |  |  |
|  | (0.108) |  |  |  |  |  |  |  |
| Lnyield_wheat (t-3~t-1)***suit**wt |  | -0.291*** |  | -0.275*** |  |  |  |  |
|  |  | (0.093) |  | (0.100) |  |  |  |  |
| Lnyield_maize (t-3~t-1)***suit**mz |  |  | -0.082 | -0.037 |  |  |  |  |
|  |  |  | (0.078) | (0.080) |  |  |  |  |
| Amount (t-3~t-1)***suit**aver |  |  |  |  | -0.112 |  |  |  |
|  |  |  |  |  | (0.078) |  |  |  |
| Num_wheat (t-3~t-1)***suit**wt |  |  |  |  |  | -0.198* |  | -0.179* |
|  |  |  |  |  |  | (0.104) |  | (0.105) |
| Num_maize (t-3~t-1)***suit**mz |  |  |  |  |  |  | -0.142 | -0.095 |
|  |  |  |  |  |  |  | (0.124) | (0.124) |
| **Prenatal controls** |  |  |  |  |  |  |  |  |
| Place_assist | -0.006** | -0.007** | -0.006** | -0.007** | -0.006** | -0.006** | -0.006** | -0.006** |
|  | (0.003) | (0.003) | (0.003) | (0.003) | (0.003) | (0.003) | (0.003) | (0.003) |
| Pregnant_check | -0.006* | -0.007* | -0.006* | -0.007* | -0.006* | -0.006* | -0.006* | -0.006* |
|  | (0.003) | (0.003) | (0.003) | (0.003) | (0.003) | (0.003) | (0.003) | (0.003) |
| Controls | Yes | Yes | Yes | Yes | Yes | Yes | Yes | Yes |
| County baseline*Time Trend | Yes | Yes | Yes | Yes | Yes | Yes | Yes | Yes |
| Prefecture FE | Yes | Yes | Yes | Yes | Yes | Yes | Yes | Yes |
| Year FE | Yes | Yes | Yes | Yes | Yes | Yes | Yes | Yes |
| Province year FE | Yes | Yes | Yes | Yes | Yes | Yes | Yes | Yes |
| Observations | 65,362 | 65,362 | 65,362 | 65,362 | 65,362 | 65,362 | 65,362 | 65,362 |
| R-squared | 0.042 | 0.042 | 0.042 | 0.042 | 0.042 | 0.042 | 0.042 | 0.042 |

Notes: ***Significant at 1%, **Significant at 5%, *Significant at 10 %. Prefecture fixed effects, year fixed effects and Province year fixed effects are included. Standard errors clustered by county in parentheses, unless otherwise indicated. Column (1) to column (6) include control variables proxied with the prenatal examination like whether the respondent mother received pre-natal doctor care and whether the child is delivered at home, hospital or clinic is assisted by doctor, nurse or midwife. County baseline controls include grain production, school number, teacher number, GDP, industry ratio, service ratio, family savings, fixed asset, government expenditure and population density.

Table S4. The coefficients of HYVs on the infant mortality from 1957-1987 using province year time trend of rural sample

|  | Whether infant died (1=yes, 0=no) | | | | | | | |
| --- | --- | --- | --- | --- | --- | --- | --- | --- |
|  | (1) | (2) | (3) | (4) | (5) | (6) | (7) | (8) |
| Lnyield_aver (t-3~t-1)***suit**aver | -0.192* |  |  |  |  |  |  |  |
|  | (0.108) |  |  |  |  |  |  |  |
| Lnyield_wheat (t-3~t-1)***suit**wt |  | -0.291*** |  | -0.273*** |  |  |  |  |
|  |  | (0.093) |  | (0.100) |  |  |  |  |
| Lnyield_maize (t-3~t-1)***suit**mz |  |  | -0.087 | -0.042 |  |  |  |  |
|  |  |  | (0.078) | (0.080) |  |  |  |  |
| Amount (t-3~t-1)***suit**aver |  |  |  |  | -0.098* |  |  |  |
|  |  |  |  |  | (0.054) |  |  |  |
| Num_wheat (t-3~t-1)***suit**wt |  |  |  |  |  | -0.127* |  | -0.115* |
|  |  |  |  |  |  | (0.065) |  | (0.068) |
| Num_maize (t-3~t-1)***suit**mz |  |  |  |  |  |  | -0.135 | -0.095 |
|  |  |  |  |  |  |  | (0.109) | (0.112) |
| **Prenatal controls** |  |  |  |  |  |  |  |  |
| Place_assist | -0.007** | -0.007** | -0.007** | -0.007** | -0.007** | -0.007** | -0.006** | -0.007** |
|  | (0.003) | (0.003) | (0.003) | (0.003) | (0.003) | (0.003) | (0.003) | (0.003) |
| Pregnant_check | -0.006* | -0.006* | -0.006* | -0.007* | -0.006* | -0.006* | -0.006* | -0.006* |
|  | (0.003) | (0.003) | (0.003) | (0.003) | (0.003) | (0.003) | (0.003) | (0.003) |
| Controls | Yes | Yes | Yes | Yes | Yes | Yes | Yes | Yes |
| County baseline*Time Trend | Yes | Yes | Yes | Yes | Yes | Yes | Yes | Yes |
| Prefecture FE | Yes | Yes | Yes | Yes | Yes | Yes | Yes | Yes |
| Year FE | Yes | Yes | Yes | Yes | Yes | Yes | Yes | Yes |
| Province year time trend | Yes | Yes | Yes | Yes | Yes | Yes | Yes | Yes |
| Observations | 65,362 | 65,362 | 65,362 | 65,362 | 65,362 | 65,362 | 65,362 | 65,362 |
| R-squared | 0.039 | 0.039 | 0.039 | 0.039 | 0.039 | 0.039 | 0.039 | 0.039 |

Notes: ***Significant at 1%, **Significant at 5%, *Significant at 10 %. Prefecture fixed effects, year fixed effects and Province year time trend are included. Standard errors clustered by county in parentheses, unless otherwise indicated. Column (1) to column (6) include control variables proxied with the prenatal examination like whether the respondent mother received pre-natal doctor care and whether the child is delivered at home, hospital or clinic is assisted by doctor, nurse or midwife. County baseline controls include grain production, school number, teacher number, GDP, industry ratio, service ratio, family savings, fixed asset, government expenditure and population density.

Table S5. Mechanism analysis: the effects of HYVs on the grain output of 1954-1966 using rural sample

|  | Ln (Grain output) | | | | | | | |
| --- | --- | --- | --- | --- | --- | --- | --- | --- |
|  | (1) | (2) | (3) | (4) | (5) | (6) | (7) | (8) |
| **Panel A** |  |  |  |  |  |  |  |  |
| Lnyield_aver (t-1)***suit**aver | 2.467 |  |  |  |  |  |  |  |
|  | (1.632) |  |  |  |  |  |  |  |
| Lnyield_wheat (t-1)***suit**wt |  | 3.035** |  | 3.309 |  |  |  |  |
|  |  | (1.439) |  | (2.062) |  |  |  |  |
| Lnyield_maize (t-1)***suit**mz |  |  | 1.203 | -0.272 |  |  |  |  |
|  |  |  | (1.328) | (1.727) |  |  |  |  |
| Amount (t-1)***suit**aver |  |  |  |  | 0.128 |  |  |  |
|  |  |  |  |  | (1.006) |  |  |  |
| Num_wheat (t-1)***suit**wt |  |  |  |  |  | 2.172* |  | 1.526 |
|  |  |  |  |  |  | (1.202) |  | (1.271) |
| Num_maize (t-1)***suit**mz |  |  |  |  |  |  | 12.256*** | 11.622** |
|  |  |  |  |  |  |  | (4.304) | (4.420) |
| Observations | 676 | 676 | 676 | 676 | 676 | 676 | 676 | 676 |
| R-squared | 0.864 | 0.864 | 0.863 | 0.864 | 0.862 | 0.863 | 0.864 | 0.864 |
| **Panel B**  **Exclude famine years** |  |  |  |  |  |  |  |  |
| Lnyield_aver (t-1)***suit**aver | 2.387 |  |  |  |  |  |  |  |
|  | (1.521) |  |  |  |  |  |  |  |
| Lnyield_wheat (t-1)***suit**wt |  | 3.113** |  | 3.388 |  |  |  |  |
|  |  | (1.393) |  | (2.084) |  |  |  |  |
| Lnyield_maize (t-1)***suit**mz |  |  | 1.220 | -0.275 |  |  |  |  |
|  |  |  | (1.322) | (1.767) |  |  |  |  |
| Amount (t-1)***suit**aver |  |  |  |  | -0.273 |  |  |  |
|  |  |  |  |  | (1.161) |  |  |  |
| Num_wheat (t-1)***suit**wt |  |  |  |  |  | 2.321* |  | 2.169 |
|  |  |  |  |  |  | (1.287) |  | (1.365) |
| Num_maize (t-1)***suit**mz |  |  |  |  |  |  | 3.809 | 2.856 |
|  |  |  |  |  |  |  | (4.317) | (4.421) |
| Controls | Yes | Yes | Yes | Yes | Yes | Yes | Yes | Yes |
| County baseline*Time Trend | Yes | Yes | Yes | Yes | Yes | Yes | Yes | Yes |
| Prefecture FE | Yes | Yes | Yes | Yes | Yes | Yes | Yes | Yes |
| Year FE | Yes | Yes | Yes | Yes | Yes | Yes | Yes | Yes |
| Province year FE | Yes | Yes | Yes | Yes | Yes | Yes | Yes | Yes |
| Observations | 495 | 495 | 495 | 495 | 495 | 495 | 495 | 495 |
| R-squared | 0.883 | 0.884 | 0.882 | 0.884 | 0.881 | 0.882 | 0.881 | 0.882 |

Notes: ***Significant at 1%, **Significant at 5%, *Significant at 10 %. Prefecture fixed effects, year fixed effects and Province year fixed effects are included. Standard errors clustered by county in parentheses.

Table S6. Mechanism analysis: the effects of HYVs on infants’ breastfeeding using rural sample

|  | Whether infant is breastfeed (1=yes, 0=no) | | | | | | | |
| --- | --- | --- | --- | --- | --- | --- | --- | --- |
|  | (1) | (2) | (3) | (4) | (5) | (6) | (7) | (8) |
| Lnyield_aver (t-3~t-1)***suit**aver | 0.444*** |  |  |  |  |  |  |  |
|  | (0.130) |  |  |  |  |  |  |  |
| Lnyield_wheat (t-3~t-1)***suit**wt |  | 0.423*** |  | 0.370*** |  |  |  |  |
|  |  | (0.121) |  | (0.119) |  |  |  |  |
| Lnyield_maize (t-3~t-1)***suit**mz |  |  | 0.182* | 0.121 |  |  |  |  |
|  |  |  | (0.093) | (0.089) |  |  |  |  |
| Amount (t-3~t-1)***suit**aver |  |  |  |  | 0.314** |  |  |  |
|  |  |  |  |  | (0.147) |  |  |  |
| Num_wheat (t-3~t-1)***suit**wt |  |  |  |  |  | 0.603*** |  | 0.612*** |
|  |  |  |  |  |  | (0.148) |  | (0.154) |
| Num_maize (t-3~t-1)***suit**mz |  |  |  |  |  |  | 0.111 | -0.048 |
|  |  |  |  |  |  |  | (0.281) | (0.254) |
| Observations | 65,362 | 65,362 | 65,362 | 65,362 | 65,362 | 65,362 | 65,362 | 65,362 |
| R-squared | 0.322 | 0.322 | 0.322 | 0.322 | 0.322 | 0.322 | 0.321 | 0.322 |
|  | Log (Month of breastfeed + 1) | | | | | | | |
| Lnyield_aver (t-3~t-1)***suit**aver | 0.803 |  |  |  |  |  |  |  |
|  | (0.613) |  |  |  |  |  |  |  |
| Lnyield_wheat (t-3~t-1)***suit**wt |  | 1.750*** |  | 1.930*** |  |  |  |  |
|  |  | (0.668) |  | (0.674) |  |  |  |  |
| Lnyield_maize (t-3~t-1)***suit**mz |  |  | -0.101 | -0.416 |  |  |  |  |
|  |  |  | (0.377) | (0.379) |  |  |  |  |
| Amount (t-3~t-1)***suit**aver |  |  |  |  | 0.360 |  |  |  |
|  |  |  |  |  | (0.542) |  |  |  |
| Num_wheat (t-3~t-1)***suit**wt |  |  |  |  |  | 1.725*** |  | 1.947*** |
|  |  |  |  |  |  | (0.564) |  | (0.606) |
| Num_maize (t-3~t-1)***suit**mz |  |  |  |  |  |  | -0.602 | -1.107 |
|  |  |  |  |  |  |  | (0.976) | (0.934) |
| Controls | Yes | Yes | Yes | Yes | Yes | Yes | Yes | Yes |
| County baseline*Time Trend | Yes | Yes | Yes | Yes | Yes | Yes | Yes | Yes |
| Prefecture FE | Yes | Yes | Yes | Yes | Yes | Yes | Yes | Yes |
| Year FE | Yes | Yes | Yes | Yes | Yes | Yes | Yes | Yes |
| Province year FE | Yes | Yes | Yes | Yes | Yes | Yes | Yes | Yes |
| Observations | 65,362 | 65,362 | 65,362 | 65,362 | 65,362 | 65,362 | 65,362 | 65,362 |
| R-squared | 0.288 | 0.288 | 0.288 | 0.288 | 0.288 | 0.288 | 0.288 | 0.288 |

Notes: ***Significant at 1%, **Significant at 5%, *Significant at 10 %. Prefecture fixed effects, year fixed effects and Province year fixed effects are included. Standard errors clustered by county in parentheses, unless otherwise indicated.

Table S7. Mechanism analysis: Childbearing decision proxied by mother age at infant’s birth year using rural sample

|  | Mother’s age at infant’s birth year | | | | | | | |
| --- | --- | --- | --- | --- | --- | --- | --- | --- |
|  | (1) | (2) | (3) | (4) | (5) | (6) | (7) | (8) |
| Lnyield_aver (t-3~t-1)***suit**aver | -1.472 |  |  |  |  |  |  |  |
|  | (2.110) |  |  |  |  |  |  |  |
| Lnyield_wheat (t-3~t-1)***suit**wt |  | 0.056 |  | 0.523 |  |  |  |  |
|  |  | (2.201) |  | (2.393) |  |  |  |  |
| Lnyield_maize (t-3~t-1)***suit**mz |  |  | -0.999 | -1.084 |  |  |  |  |
|  |  |  | (1.417) | (1.532) |  |  |  |  |
| Amount (t-3~t-1)***suit**aver |  |  |  |  | 1.527 |  |  |  |
|  |  |  |  |  | (1.425) |  |  |  |
| Num_wheat (t-3~t-1)***suit**wt |  |  |  |  |  | 2.056 |  | 1.422 |
|  |  |  |  |  |  | (2.112) |  | (2.212) |
| Num_maize (t-3~t-1)***suit**mz |  |  |  |  |  |  | 3.548 | 3.179 |
|  |  |  |  |  |  |  | (2.184) | (2.242) |
| Controls | Yes | Yes | Yes | Yes | Yes | Yes | Yes | Yes |
| County baseline*Time Trend | Yes | Yes | Yes | Yes | Yes | Yes | Yes | Yes |
| Prefecture FE | Yes | Yes | Yes | Yes | Yes | Yes | Yes | Yes |
| Year FE | Yes | Yes | Yes | Yes | Yes | Yes | Yes | Yes |
| Province year FE | Yes | Yes | Yes | Yes | Yes | Yes | Yes | Yes |
| Observations | 65,362 | 65,362 | 65,362 | 65,362 | 65,362 | 65,362 | 65,362 | 65,362 |
| R-squared | 0.723 | 0.723 | 0.723 | 0.723 | 0.723 | 0.723 | 0.723 | 0.723 |

Notes: ***Significant at 1%, **Significant at 5%, *Significant at 10 %. Prefecture fixed effects, year fixed effects and Province year fixed effects are included. Standard errors clustered by county in parentheses, unless otherwise indicated.

Table S8. The coefficients of HYVs on the infant mortality from 1956-1987 using the moving average of lag two year of rural sample

|  | Whether infant died (1=yes, 0=no) | | | | | | | |
| --- | --- | --- | --- | --- | --- | --- | --- | --- |
|  | (1) | (2) | (3) | (4) | (5) | (6) | (7) | (8) |
| Lnyield_aver (t-2~t-1)***suit**aver | -0.206* |  |  |  |  |  |  |  |
|  | (0.108) |  |  |  |  |  |  |  |
| Lnyield_wheat (t-2~t-1)***suit**wt |  | -0.283*** |  | -0.268*** |  |  |  |  |
|  |  | (0.094) |  | (0.100) |  |  |  |  |
| Lnyield_maize (t-2~t-1)***suit**mz |  |  | -0.079 | -0.036 |  |  |  |  |
|  |  |  | (0.078) | (0.080) |  |  |  |  |
| Amount (t-2~t-1)***suit**aver |  |  |  |  | -0.083 |  |  |  |
|  |  |  |  |  | (0.077) |  |  |  |
| Num_wheat (t-2~t-1)***suit**wt |  |  |  |  |  | -0.170 |  | -0.155 |
|  |  |  |  |  |  | (0.103) |  | (0.104) |
| Num_maize (t-2~t-1)***suit**mz |  |  |  |  |  |  | -0.106 | -0.071 |
|  |  |  |  |  |  |  | (0.107) | (0.107) |
| **Prenatal controls** |  |  |  |  |  |  |  |  |
| Place_assist | -0.007** | -0.007** | -0.006** | -0.007** | -0.006** | -0.007** | -0.006** | -0.007** |
|  | (0.003) | (0.003) | (0.003) | (0.003) | (0.003) | (0.003) | (0.003) | (0.003) |
| Pregnant_check | -0.007* | -0.007* | -0.006* | -0.007* | -0.006* | -0.006* | -0.006* | -0.006* |
|  | (0.003) | (0.003) | (0.003) | (0.003) | (0.003) | (0.003) | (0.003) | (0.003) |
| Controls | Yes | Yes | Yes | Yes | Yes | Yes | Yes | Yes |
| County baseline*Time Trend | Yes | Yes | Yes | Yes | Yes | Yes | Yes | Yes |
| Prefecture FE | Yes | Yes | Yes | Yes | Yes | Yes | Yes | Yes |
| Year FE | Yes | Yes | Yes | Yes | Yes | Yes | Yes | Yes |
| Province year FE | Yes | Yes | Yes | Yes | Yes | Yes | Yes | Yes |
| Observations | 65,497 | 65,497 | 65,497 | 65,497 | 65,497 | 65,497 | 65,497 | 65,497 |
| R-squared | 0.042 | 0.043 | 0.042 | 0.043 | 0.042 | 0.042 | 0.042 | 0.042 |

Notes: ***Significant at 1%, **Significant at 5%, *Significant at 10 %. Prefecture fixed effects, year fixed effects and Province year fixed effects are included. Standard errors clustered by county in parentheses, unless otherwise indicated. Column (1) to column (8) include control variables proxied with the prenatal examination like whether the respondent mother received pre-natal doctor care and whether the child is delivered at home, hospital or clinic is assisted by doctor, nurse or midwife. County baseline controls include grain production, school number, teacher number, GDP, industry ratio, service ratio, family savings, fixed asset, government expenditure and population density.

Table S9. The coefficients of HYVs on the infant mortality from 1959-1987 using the moving average of lag five year of rural sample

|  | Whether infant died (1=yes, 0=no) | | | | | | | |
| --- | --- | --- | --- | --- | --- | --- | --- | --- |
|  | (1) | (2) | (3) | (4) | (5) | (6) | (7) | (8) |
| Lnyield_aver (t-5~t-1)***suit**aver | -0.208* |  |  |  |  |  |  |  |
|  | (0.108) |  |  |  |  |  |  |  |
| Lnyield_wheat (t-5~t-1)***suit**wt |  | -0.291*** |  | -0.276*** |  |  |  |  |
|  |  | (0.092) |  | (0.099) |  |  |  |  |
| Lnyield_maize (t-5~t-1)***suit**mz |  |  | -0.079 | -0.034 |  |  |  |  |
|  |  |  | (0.078) | (0.080) |  |  |  |  |
| Amount (t-5~t-1)***suit**aver |  |  |  |  | -0.127 |  |  |  |
|  |  |  |  |  | (0.090) |  |  |  |
| Num_wheat (t-5~t-1)***suit**wt |  |  |  |  |  | -0.213* |  | -0.196* |
|  |  |  |  |  |  | (0.117) |  | (0.118) |
| Num_maize (t-5~t-1)***suit**mz |  |  |  |  |  |  | -0.143 | -0.085 |
|  |  |  |  |  |  |  | (0.143) | (0.144) |
| **Prenatal controls** |  |  |  |  |  |  |  |  |
| Place_assist | -0.006** | -0.007** | -0.006** | -0.007** | -0.006** | -0.006** | -0.006** | -0.006** |
|  | (0.003) | (0.003) | (0.003) | (0.003) | (0.003) | (0.003) | (0.003) | (0.003) |
| Pregnant_check | -0.006* | -0.006* | -0.006* | -0.006* | -0.006* | -0.006* | -0.006* | -0.006* |
|  | (0.003) | (0.003) | (0.003) | (0.003) | (0.003) | (0.003) | (0.003) | (0.003) |
| Controls | Yes | Yes | Yes | Yes | Yes | Yes | Yes | Yes |
| County baseline*Time Trend | Yes | Yes | Yes | Yes | Yes | Yes | Yes | Yes |
| Prefecture FE | Yes | Yes | Yes | Yes | Yes | Yes | Yes | Yes |
| Year FE | Yes | Yes | Yes | Yes | Yes | Yes | Yes | Yes |
| Province year FE | Yes | Yes | Yes | Yes | Yes | Yes | Yes | Yes |
| Observations | 102,219 | 102,219 | 102,219 | 102,219 | 102,219 | 102,219 | 102,219 | 102,219 |
| R-squared | 0.041 | 0.041 | 0.041 | 0.041 | 0.041 | 0.041 | 0.041 | 0.041 |

Notes: ***Significant at 1%, **Significant at 5%, *Significant at 10 %. Prefecture fixed effects, year fixed effects and Province year fixed effects are included. Standard errors clustered by county in parentheses, unless otherwise indicated. Column (1) to column (8) include control variables proxied with the prenatal examination like whether the respondent mother received pre-natal doctor care and whether the child is delivered at home, hospital or clinic is assisted by doctor, nurse or midwife. County baseline controls include grain production, school number, teacher number, GDP, industry ratio, service ratio, family savings, fixed asset, government expenditure and population density.

Table S10. The coefficients of HYVs on the infant mortality from 1957-1987 using rural sample (Exclude the child who died during the first week after birth)

|  | Whether infant died after the first week (1=yes, 0=no) | | | | | | | |
| --- | --- | --- | --- | --- | --- | --- | --- | --- |
|  | (1) | (2) | (3) | (4) | (5) | (6) | (7) | (8) |
| Lnyield_aver (t-3~t-1)***suit**aver | -0.115 |  |  |  |  |  |  |  |
|  | (0.076) |  |  |  |  |  |  |  |
| Lnyield_wheat (t-3~t-1)***suit**wt |  | -0.117* |  | -0.103 |  |  |  |  |
|  |  | (0.067) |  | (0.068) |  |  |  |  |
| Lnyield_maize (t-3~t-1)***suit**mz |  |  | -0.048 | -0.031 |  |  |  |  |
|  |  |  | (0.050) | (0.050) |  |  |  |  |
| Amount (t-3~t-1)***suit**aver |  |  |  |  | -0.023 |  |  |  |
|  |  |  |  |  | (0.048) |  |  |  |
| Num_wheat (t-3~t-1)***suit**wt |  |  |  |  |  | -0.051 |  | -0.056 |
|  |  |  |  |  |  | (0.068) |  | (0.068) |
| Num_maize (t-3~t-1)***suit**mz |  |  |  |  |  |  | 0.008 | 0.022 |
|  |  |  |  |  |  |  | (0.071) | (0.072) |
| **Prenatal controls** |  |  |  |  |  |  |  |  |
| Place_assist | -0.004** | -0.004** | -0.004** | -0.004** | -0.004** | -0.004** | -0.004** | -0.004** |
|  | (0.002) | (0.002) | (0.002) | (0.002) | (0.002) | (0.002) | (0.002) | (0.002) |
| Pregnant_check | -0.005** | -0.005** | -0.005** | -0.005** | -0.005** | -0.005** | -0.005** | -0.005** |
|  | (0.002) | (0.002) | (0.002) | (0.002) | (0.002) | (0.002) | (0.002) | (0.002) |
| Controls | Yes | Yes | Yes | Yes | Yes | Yes | Yes | Yes |
| County baseline*Time Trend | Yes | Yes | Yes | Yes | Yes | Yes | Yes | Yes |
| Prefecture FE | Yes | Yes | Yes | Yes | Yes | Yes | Yes | Yes |
| Year FE | Yes | Yes | Yes | Yes | Yes | Yes | Yes | Yes |
| Province year FE | Yes | Yes | Yes | Yes | Yes | Yes | Yes | Yes |
| Observations | 65,362 | 65,362 | 65,362 | 65,362 | 65,362 | 65,362 | 65,362 | 65,362 |
| R-squared | 0.026 | 0.026 | 0.026 | 0.026 | 0.026 | 0.026 | 0.026 | 0.026 |

Notes: ***Significant at 1%, **Significant at 5%, *Significant at 10 %. Prefecture fixed effects, year fixed effects and Province year fixed effects are included. Standard errors clustered by county in parentheses, unless otherwise indicated. Column (1) to column (6) include control variables proxied with the prenatal examination like whether the respondent mother received pre-natal doctor care and whether the child is delivered at home, hospital or clinic is assisted by doctor, nurse or midwife. County baseline controls include grain production, school number, teacher number, GDP, industry ratio, service ratio, family savings, fixed asset, government expenditure and population density.

Table S11. The coefficients of HYVs on the infant mortality from 1957-1987 using rural sample (Exclude famine years)

|  | Whether infant died (1=yes, 0=no) | | | | | | | |
| --- | --- | --- | --- | --- | --- | --- | --- | --- |
|  | (1) | (2) | (3) | (4) | (5) | (6) | (7) | (8) |
| Lnyield_aver (t-3~t-1)***suit**aver | -0.179* |  |  |  |  |  |  |  |
|  | (0.104) |  |  |  |  |  |  |  |
| Lnyield_wheat (t-3~t-1)***suit**wt |  | -0.276*** |  | -0.270*** |  |  |  |  |
|  |  | (0.090) |  | (0.098) |  |  |  |  |
| Lnyield_maize (t-3~t-1)***suit**mz |  |  | -0.058 | -0.014 |  |  |  |  |
|  |  |  | (0.075) | (0.078) |  |  |  |  |
| Amount (t-3~t-1)***suit**aver |  |  |  |  | -0.102 |  |  |  |
|  |  |  |  |  | (0.075) |  |  |  |
| Num_wheat (t-3~t-1)***suit**wt |  |  |  |  |  | -0.205** |  | -0.188* |
|  |  |  |  |  |  | (0.099) |  | (0.100) |
| Num_maize (t-3~t-1)***suit**mz |  |  |  |  |  |  | -0.134 | -0.085 |
|  |  |  |  |  |  |  | (0.121) | (0.120) |
| **Prenatal controls** |  |  |  |  |  |  |  |  |
| Place_assist | -0.006** | -0.006** | -0.006* | -0.006** | -0.006** | -0.006** | -0.006* | -0.006** |
|  | (0.003) | (0.003) | (0.003) | (0.003) | (0.003) | (0.003) | (0.003) | (0.003) |
| Pregnant_check | -0.006* | -0.006* | -0.006* | -0.006* | -0.006* | -0.006* | -0.006* | -0.006* |
|  | (0.004) | (0.004) | (0.004) | (0.004) | (0.004) | (0.004) | (0.004) | (0.004) |
| Controls | Yes | Yes | Yes | Yes | Yes | Yes | Yes | Yes |
| County baseline*Time Trend | Yes | Yes | Yes | Yes | Yes | Yes | Yes | Yes |
| Prefecture FE | Yes | Yes | Yes | Yes | Yes | Yes | Yes | Yes |
| Year FE | Yes | Yes | Yes | Yes | Yes | Yes | Yes | Yes |
| Province year FE | Yes | Yes | Yes | Yes | Yes | Yes | Yes | Yes |
| Observations | 63,777 | 63,777 | 63,777 | 63,777 | 63,777 | 63,777 | 63,777 | 63,777 |
| R-squared | 0.038 | 0.039 | 0.038 | 0.039 | 0.038 | 0.038 | 0.038 | 0.038 |

Notes: ***Significant at 1%, **Significant at 5%, *Significant at 10 %. Prefecture fixed effects, year fixed effects and Province year fixed effects are included. Standard errors clustered by county in parentheses, unless otherwise indicated. Column (1) to column (6) include control variables proxied with the prenatal examination like whether the respondent mother received pre-natal doctor care and whether the child is delivered at home, hospital or clinic is assisted by doctor, nurse or midwife. County baseline controls include grain production, school number, teacher number, GDP, industry ratio, service ratio, family savings, fixed asset, government expenditure and population density.

Table S12. The correlation between famine with pre-famine and post-famine productivity at the county level

|  | Excess death rate (1959-1961) | | | | Lnyield_aver (1962)***suit**aver | Lnyield_aver (1962~1964)***suit**aver | Amount (1962)***suit**aver | Amount (1962~1964)***suit**aver |
| --- | --- | --- | --- | --- | --- | --- | --- | --- |
|  | (1) | (2) | (3) | (4) | (5) | (6) | (7) | (8) |
|  | **Pre-famine** | | | | **Post-famine** | | | |
| Lnyield_aver (1958)***suit**aver | -2.076 |  |  |  |  |  |  |  |
|  | (10.684) |  |  |  |  |  |  |  |
| Lnyield_aver (1956~1958)***suit**aver |  | -1.761 |  |  |  |  |  |  |
|  |  | (7.238) |  |  |  |  |  |  |
| Amount (1958)***suit**aver |  |  | 10.802 |  |  |  |  |  |
|  |  |  | (12.676) |  |  |  |  |  |
| Amount (1956~1958)***suit**aver |  |  |  | -0.456 |  |  |  |  |
|  |  |  |  | (9.030) |  |  |  |  |
| Excess death rate |  |  |  |  | -0.000 | -0.000 | 0.000 | -0.000 |
|  |  |  |  |  | (0.000) | (0.000) | (0.000) | (0.000) |
| Controls | Yes | Yes | Yes | Yes | Yes | Yes | Yes | Yes |
| County baseline*Time Trend | Yes | Yes | Yes | Yes | Yes | Yes | Yes | Yes |
| Prefecture FE | Yes | Yes | Yes | Yes | Yes | Yes | Yes | Yes |
| Year FE | Yes | Yes | Yes | Yes | Yes | Yes | Yes | Yes |
| Province year time trend | Yes | Yes | Yes | Yes | Yes | Yes | Yes | Yes |
| Observations | 344 | 211 | 344 | 211 | 388 | 388 | 388 | 388 |
| R-squared | 0.892 | 0.921 | 0.892 | 0.921 | 0.929 | 0.927 | 0.990 | 0.975 |

Notes: ***Significant at 1%, **Significant at 5%, *Significant at 10 %. Prefecture fixed effects, year fixed effects and Province year time trend are included. Standard errors clustered by county in parentheses, unless otherwise indicated. County baseline controls include grain production, school number, teacher number, GDP, industry ratio, service ratio, family savings, fixed asset, government expenditure and population density. For that the Famine occurs between 1959 and 1961, we take the year 1958 as the pre-famine year. Also, we employ the average value of Lnyield_aver and Amount from 1956 to 1958 as a robustness check. Accordingly, we take the year 1962 as the post-famine year. In addition, we employ the average value of Lnyield_aver and Amount from 1962 to 1964 as a robustness check. We use the excess death rate as the indicator of the severity of the famine.

Table S13. The coefficients of HYVs on the infant mortality from 1957-1987 using rural sample excluding *Shanghai* and *Guizhou* province

|  | Whether infant died (1=yes, 0=no) | | | | | | | |
| --- | --- | --- | --- | --- | --- | --- | --- | --- |
|  | (1) | (2) | (3) | (4) | (5) | (6) | (7) | (8) |
| Lnyield_aver (t-3~t-1)***suit**aver | -0.366*** |  |  |  |  |  |  |  |
|  | (0.129) |  |  |  |  |  |  |  |
| Lnyield_wheat (t-3~t-1)***suit**wt |  | -0.307** |  | -0.238* |  |  |  |  |
|  |  | (0.129) |  | (0.140) |  |  |  |  |
| Lnyield_maize (t-3~t-1)***suit**mz |  |  | -0.155* | -0.115 |  |  |  |  |
|  |  |  | (0.081) | (0.086) |  |  |  |  |
| Amount (t-3~t-1)***suit**aver |  |  |  |  | -0.139* |  |  |  |
|  |  |  |  |  | (0.084) |  |  |  |
| Num_wheat (t-3~t-1)***suit**wt |  |  |  |  |  | -0.180 |  | -0.155 |
|  |  |  |  |  |  | (0.122) |  | (0.124) |
| Num_maize (t-3~t-1)***suit**mz |  |  |  |  |  |  | -0.152 | -0.112 |
|  |  |  |  |  |  |  | (0.129) | (0.131) |
| **Prenatal controls** |  |  |  |  |  |  |  |  |
| Place_assist | -0.007** | -0.008** | -0.007** | -0.007** | -0.007** | -0.007** | -0.007** | -0.007** |
|  | (0.003) | (0.003) | (0.003) | (0.003) | (0.003) | (0.003) | (0.003) | (0.003) |
| Pregnant_check | -0.008** | -0.007** | -0.008** | -0.008** | -0.007** | -0.007** | -0.007** | -0.007** |
|  | (0.003) | (0.003) | (0.003) | (0.003) | (0.003) | (0.003) | (0.003) | (0.003) |
| Controls | Yes | Yes | Yes | Yes | Yes | Yes | Yes | Yes |
| County baseline*Time Trend | Yes | Yes | Yes | Yes | Yes | Yes | Yes | Yes |
| Prefecture FE | Yes | Yes | Yes | Yes | Yes | Yes | Yes | Yes |
| Year FE | Yes | Yes | Yes | Yes | Yes | Yes | Yes | Yes |
| Province year time trend | Yes | Yes | Yes | Yes | Yes | Yes | Yes | Yes |
| Observations | 46,041 | 46,041 | 46,041 | 46,041 | 46,041 | 46,041 | 46,041 | 46,041 |
| R-squared | 0.038 | 0.037 | 0.037 | 0.038 | 0.037 | 0.037 | 0.037 | 0.037 |

Notes: ***Significant at 1%, **Significant at 5%, *Significant at 10 %. Prefecture fixed effects, year fixed effects and Province year time trend are included. Standard errors clustered by county in parentheses, unless otherwise indicated. Column (1) to column (6) include control variables proxied with the prenatal examination like whether the respondent mother received pre-natal doctor care and whether the child is delivered at home, hospital or clinic is assisted by doctor, nurse or midwife. County baseline controls include grain production, school number, teacher number, GDP, industry ratio, service ratio, family savings, fixed asset, government expenditure and population density.

Table S14. Descriptive statistics using the whole sample

| Variable Definition | Obs | Mean | Std. Dev. | Min | Max |
| --- | --- | --- | --- | --- | --- |
| ***Dependent variable*** |  |  |  |  |  |
| Whether infant died | 106,714 | 0.055 | 0.227 | 0 | 1 |
| ***Independent variable*** |  |  |  |  |  |
| Lnyield_wheat | 106,714 | 6.065 | 0.220 | 5.037 | 6.516 |
| Lnyield_maize | 106,714 | 6.275 | 0.309 | 5.170 | 6.686 |
| Lnyield_aver | 106,714 | 6.199 | 0.231 | 5.047 | 6.614 |
| Num_wheat | 106,714 | 4.075 | 3.718 | 0 | 17 |
| Num_maize | 106,714 | 1.587 | 2.277 | 0 | 14 |
| Amount | 106,714 | 5.662 | 4.780 | 0 | 22 |
| Suit_wheat | 106,714 | 0.002 | 0.010 | -0.022 | 0.032 |
| Suit_maize | 106,714 | 0.001 | 0.009 | -0.014 | 0.024 |
| Suit_aver | 106,714 | 0.002 | 0.008 | -0.013 | 0.020 |
| ***Control variable*** |  |  |  |  |  |
| *Individual level* |  |  |  |  |  |
| Child Male | 106,714 | 0.524 | 0.499 | 0 | 1 |
| Birth Order Number | 106,714 | 2.303 | 1.522 | 1 | 15 |
| Child Multiple | 106,714 | 0.009 | 0.096 | 0 | 1 |
| Boy before | 106,714 | 0.411 | 0.492 | 0 | 1 |
| Birth weight | 106,714 | 6.448 | 1.075 | 4 | 9 |
| Mother edu | 106,714 | 0.006 | 0.078 | 0 | 1 |
| Mother work | 106,714 | 0.758 | 0.428 | 0 | 1 |
| Mother age at childbirth | 106,714 | 25.720 | 4.350 | 16 | 47 |
| Father age at childbirth | 106,714 | 28.325 | 5.153 | 16 | 60 |
| Wealth_index | 106,714 | -0.001 | 0.841 | -1.77 | 29.81 |
| Urban | 106,714 | 0.278 | 0.448 | 0 | 1 |
| Place_assist | 106,714 | 0.367 | 0.482 | 0 | 1 |
| Pregnant_check | 106,714 | 0.193 | 0.394 | 0 | 1 |
| *County level* |  |  |  |  |  |
| Grain production | 233 | 0.284 | 0.230 | 0.001 | 1.471 |
| Teacher number | 230 | 2405.874 | 3000.438 | 81.000 | 18502.000 |
| School number | 231 | 429.069 | 539.395 | 16.000 | 3980.000 |
| GDP | 231 | 0.060 | 0.087 | 0.002 | 0.503 |
| Industry ratio | 231 | 0.244 | 0.185 | 0.008 | 0.950 |
| Service ratio | 232 | 0.233 | 0.163 | 0.017 | 0.913 |
| Family savings | 233 | 0.031 | 0.123 | 0.000 | 0.998 |
| Fixed asset | 233 | 0.015 | 0.050 | 0.000 | 0.411 |
| Government expenditure | 230 | 0.104 | 0.131 | 0.002 | 0.671 |
| Population density | 233 | 0.288 | 0.861 | 0.001 | 4.689 |
| Average temperature | 106,714 | 11.275 | 3.590 | -18 | 29.6 |
| Average precipitation | 106,714 | 499.557 | 267.173 | 0 | 3270 |
| *Province level* |  |  |  |  |  |
| Cultivated area | 106,714 | 1.062 | 0.635 | 0.074 | 3.318 |
| Quota | 106,714 | 10.508 | 12.778 | -19.4 | 47.68 |
| Ln_gdp | 106,714 | 5.835 | 0.832 | 4.290 | 8.246 |

Notes: The data sources from which the variables come from are China In-depth Fertility Sample Surveys (1985 and 1987) and High-yield varieties (HYVs) dataset. The county level statistics are hand collected by ourselves from *Wanfang Local Chronicles Knowledge Service Platform* and *China Economic and Social Big Data Research Platform*, which are only observed in the baseline. The observations of the county level variables are equal to the number of the county.

Table S15. The coefficients of HYVs on the infant mortality from 1957-1987 using the whole sample

|  | Whether infant died (1=yes, 0=no) | | | | | | | |
| --- | --- | --- | --- | --- | --- | --- | --- | --- |
|  | (1) | (2) | (3) | (4) | (5) | (6) | (7) | (8) |
| Lnyield_aver (t-3~t-1)***suit**aver | -0.201** |  |  |  |  |  |  |  |
|  | (0.090) |  |  |  |  |  |  |  |
| Lnyield_wheat (t-3~t-1)***suit**wt |  | -0.254*** |  | -0.235*** |  |  |  |  |
|  |  | (0.081) |  | (0.087) |  |  |  |  |
| Lnyield_maize (t-3~t-1)***suit**mz |  |  | -0.090 | -0.052 |  |  |  |  |
|  |  |  | (0.069) | (0.072) |  |  |  |  |
| Amount (t-3~t-1)***suit**aver |  |  |  |  | -0.168** |  |  |  |
|  |  |  |  |  | (0.070) |  |  |  |
| Num_wheat (t-3~t-1)***suit**wt |  |  |  |  |  | -0.299*** |  | -0.279*** |
|  |  |  |  |  |  | (0.094) |  | (0.096) |
| Num_maize (t-3~t-1)***suit**mz |  |  |  |  |  |  | -0.176 | -0.106 |
|  |  |  |  |  |  |  | (0.118) | (0.118) |
| **Prenatal controls** |  |  |  |  |  |  |  |  |
| Place_assist | -0.011*** | -0.011*** | -0.010*** | -0.011*** | -0.011*** | -0.011*** | -0.010*** | -0.011*** |
|  | (0.002) | (0.002) | (0.002) | (0.002) | (0.002) | (0.002) | (0.002) | (0.002) |
| Pregnant_check | -0.008*** | -0.008*** | -0.008*** | -0.008*** | -0.008*** | -0.008*** | -0.008*** | -0.008*** |
|  | (0.003) | (0.003) | (0.002) | (0.003) | (0.003) | (0.003) | (0.002) | (0.003) |
| Controls | Yes | Yes | Yes | Yes | Yes | Yes | Yes | Yes |
| County baseline*Time Trend | Yes | Yes | Yes | Yes | Yes | Yes | Yes | Yes |
| Prefecture FE | Yes | Yes | Yes | Yes | Yes | Yes | Yes | Yes |
| Year FE | Yes | Yes | Yes | Yes | Yes | Yes | Yes | Yes |
| Province year FE | Yes | Yes | Yes | Yes | Yes | Yes | Yes | Yes |
| Observations | 90,578 | 90,578 | 90,578 | 90,578 | 90,578 | 90,578 | 90,578 | 90,578 |
| R-squared | 0.043 | 0.043 | 0.043 | 0.043 | 0.043 | 0.043 | 0.043 | 0.043 |

Notes: ***Significant at 1%, **Significant at 5%, *Significant at 10 %. Prefecture fixed effects, year fixed effects and Province year fixed effects are included. Standard errors clustered by county in parentheses, unless otherwise indicated. Column (1) to column (6) include control variables proxied with the prenatal examination like whether the respondent mother received pre-natal doctor care and whether the child is delivered at home, hospital or clinic is assisted by doctor, nurse or midwife. County baseline controls include grain production, school number, teacher number, GDP, industry ratio, service ratio, family savings, fixed asset, government expenditure and population density.

Table S16. The coefficients of HYVs on the infant mortality from 1957-1987 using the whole sample with weight

|  | Whether infant died (1=yes, 0=no) | | | | | | | |
| --- | --- | --- | --- | --- | --- | --- | --- | --- |
|  | (1) | (2) | (3) | (4) | (5) | (6) | (7) | (8) |
| Lnyield_aver (t-3~t-1)***suit**aver | -0.168 |  |  |  |  |  |  |  |
|  | (0.185) |  |  |  |  |  |  |  |
| Lnyield_wheat (t-3~t-1)***suit**wt |  | -0.307** |  | -0.318* |  |  |  |  |
|  |  | (0.150) |  | (0.164) |  |  |  |  |
| Lnyield_maize (t-3~t-1)***suit**mz |  |  | -0.030 | 0.028 |  |  |  |  |
|  |  |  | (0.144) | (0.148) |  |  |  |  |
| Amount (t-3~t-1)***suit**aver |  |  |  |  | -0.146 |  |  |  |
|  |  |  |  |  | (0.130) |  |  |  |
| Num_wheat (t-3~t-1)***suit**wt |  |  |  |  |  | -0.482** |  | -0.572** |
|  |  |  |  |  |  | (0.207) |  | (0.238) |
| Num_maize (t-3~t-1)***suit**mz |  |  |  |  |  |  | 0.177 | 0.348 |
|  |  |  |  |  |  |  | (0.231) | (0.251) |
| **Prenatal controls** |  |  |  |  |  |  |  |  |
| Place_assist | -0.014** | -0.015** | -0.014** | -0.015** | -0.014** | -0.015** | -0.014** | -0.015** |
|  | (0.006) | (0.006) | (0.006) | (0.006) | (0.006) | (0.006) | (0.006) | (0.006) |
| Pregnant_check | -0.018*** | -0.018*** | -0.018*** | -0.018*** | -0.018*** | -0.018*** | -0.017*** | -0.017*** |
|  | (0.006) | (0.006) | (0.006) | (0.006) | (0.006) | (0.006) | (0.006) | (0.006) |
| Controls | Yes | Yes | Yes | Yes | Yes | Yes | Yes | Yes |
| County baseline*Time Trend | Yes | Yes | Yes | Yes | Yes | Yes | Yes | Yes |
| Prefecture FE | Yes | Yes | Yes | Yes | Yes | Yes | Yes | Yes |
| Year FE | Yes | Yes | Yes | Yes | Yes | Yes | Yes | Yes |
| Province year FE | Yes | Yes | Yes | Yes | Yes | Yes | Yes | Yes |
| Observations | 90,578 | 90,578 | 90,578 | 90,578 | 90,578 | 90,578 | 90,578 | 90,578 |
| R-squared | 0.078 | 0.079 | 0.078 | 0.079 | 0.078 | 0.079 | 0.078 | 0.079 |

Notes: ***Significant at 1%, **Significant at 5%, *Significant at 10 %. Prefecture fixed effects, year fixed effects and Province year fixed effects are included. Standard errors clustered by county in parentheses, unless otherwise indicated. Column (1) to column (6) include control variables proxied with the prenatal examination like whether the respondent mother received pre-natal doctor care and whether the child is delivered at home, hospital or clinic is assisted by doctor, nurse or midwife. County baseline controls include grain production, school number, teacher number, GDP, industry ratio, service ratio, family savings, fixed asset, government expenditure and population density. The weight is calculated as follows: First, determine the number of women in different age groups for each year in the sample regions. Next, using the age-group-specific female population data from the 1953, 1964, and 1982 Censuses, divide the sample number of women in each age group by the corresponding female population in the Census data for that year. The weight for each age group is then obtained by taking the reciprocal of this ratio.

Table S17. The coefficients of HYVs on the infant mortality from 1957-1987 using the whole sample with province year time trend

|  | Whether infant died (1=yes, 0=no) | | | | | | | |
| --- | --- | --- | --- | --- | --- | --- | --- | --- |
|  | (1) | (2) | (3) | (4) | (5) | (6) | (7) | (8) |
| Lnyield_aver (t-3~t-1)***suit**aver | -0.180** |  |  |  |  |  |  |  |
|  | (0.089) |  |  |  |  |  |  |  |
| Lnyield_wheat (t-3~t-1)***suit**wt |  | -0.258*** |  | -0.237*** |  |  |  |  |
|  |  | (0.080) |  | (0.086) |  |  |  |  |
| Lnyield_maize (t-3~t-1)***suit**mz |  |  | -0.094 | -0.056 |  |  |  |  |
|  |  |  | (0.069) | (0.071) |  |  |  |  |
| Amount (t-3~t-1)***suit**aver |  |  |  |  | -0.084* |  |  |  |
|  |  |  |  |  | (0.047) |  |  |  |
| Num_wheat (t-3~t-1)***suit**wt |  |  |  |  |  | -0.110** |  | -0.097* |
|  |  |  |  |  |  | (0.055) |  | (0.057) |
| Num_maize (t-3~t-1)***suit**mz |  |  |  |  |  |  | -0.138 | -0.106 |
|  |  |  |  |  |  |  | (0.099) | (0.101) |
| **Prenatal controls** |  |  |  |  |  |  |  |  |
| Place_assist | -0.011*** | -0.011*** | -0.011*** | -0.011*** | -0.011*** | -0.011*** | -0.011*** | -0.011*** |
|  | (0.002) | (0.002) | (0.002) | (0.002) | (0.002) | (0.002) | (0.002) | (0.002) |
| Pregnant_check | -0.008*** | -0.008*** | -0.008*** | -0.008*** | -0.008*** | -0.008*** | -0.008*** | -0.008*** |
|  | (0.002) | (0.002) | (0.002) | (0.002) | (0.002) | (0.002) | (0.002) | (0.002) |
| Controls | Yes | Yes | Yes | Yes | Yes | Yes | Yes | Yes |
| County baseline*Time Trend | Yes | Yes | Yes | Yes | Yes | Yes | Yes | Yes |
| Prefecture FE | Yes | Yes | Yes | Yes | Yes | Yes | Yes | Yes |
| Year FE | Yes | Yes | Yes | Yes | Yes | Yes | Yes | Yes |
| Province year time trend | Yes | Yes | Yes | Yes | Yes | Yes | Yes | Yes |
| Observations | 90,578 | 90,578 | 90,578 | 90,578 | 90,578 | 90,578 | 90,578 | 90,578 |
| R-squared | 0.040 | 0.041 | 0.040 | 0.041 | 0.040 | 0.040 | 0.040 | 0.040 |

Notes: ***Significant at 1%, **Significant at 5%, *Significant at 10 %. Prefecture fixed effects, year fixed effects and Province year time trend are included. Standard errors clustered by county in parentheses, unless otherwise indicated. Column (1) to column (6) include control variables proxied with the prenatal examination like whether the respondent mother received pre-natal doctor care and whether the child is delivered at home, hospital or clinic is assisted by doctor, nurse or midwife. County baseline controls include grain production, school number, teacher number, GDP, industry ratio, service ratio, family savings, fixed asset, government expenditure and population density.

Table S18. The coefficients of HYVs on the infant mortality from 1957-1987 using the whole sample with province year time trend and weight

|  | Whether infant died (1=yes, 0=no) | | | | | | | |
| --- | --- | --- | --- | --- | --- | --- | --- | --- |
|  | (1) | (2) | (3) | (4) | (5) | (6) | (7) | (8) |
| Lnyield_aver (t-3~t-1)***suit**aver | -0.159 |  |  |  |  |  |  |  |
|  | (0.187) |  |  |  |  |  |  |  |
| Lnyield_wheat (t-3~t-1)***suit**wt |  | -0.306** |  | -0.301* |  |  |  |  |
|  |  | (0.154) |  | (0.165) |  |  |  |  |
| Lnyield_maize (t-3~t-1)***suit**mz |  |  | -0.068 | -0.013 |  |  |  |  |
|  |  |  | (0.145) | (0.150) |  |  |  |  |
| Amount (t-3~t-1)***suit**aver |  |  |  |  | -0.095 |  |  |  |
|  |  |  |  |  | (0.104) |  |  |  |
| Num_wheat (t-3~t-1)***suit**wt |  |  |  |  |  | -0.250* |  | -0.292** |
|  |  |  |  |  |  | (0.132) |  | (0.147) |
| Num_maize (t-3~t-1)***suit**mz |  |  |  |  |  |  | 0.124 | 0.227 |
|  |  |  |  |  |  |  | (0.207) | (0.213) |
| **Prenatal controls** |  |  |  |  |  |  |  |  |
| Place_assist | -0.015** | -0.015** | -0.015** | -0.015** | -0.015** | -0.015** | -0.015** | -0.015** |
|  | (0.006) | (0.006) | (0.006) | (0.006) | (0.006) | (0.006) | (0.006) | (0.006) |
| Pregnant_check | -0.017*** | -0.018*** | -0.017*** | -0.018*** | -0.017*** | -0.017*** | -0.017*** | -0.017*** |
|  | (0.006) | (0.006) | (0.006) | (0.006) | (0.006) | (0.006) | (0.006) | (0.006) |
| Controls | Yes | Yes | Yes | Yes | Yes | Yes | Yes | Yes |
| County baseline*Time Trend | Yes | Yes | Yes | Yes | Yes | Yes | Yes | Yes |
| Prefecture FE | Yes | Yes | Yes | Yes | Yes | Yes | Yes | Yes |
| Year FE | Yes | Yes | Yes | Yes | Yes | Yes | Yes | Yes |
| Province year time trend | Yes | Yes | Yes | Yes | Yes | Yes | Yes | Yes |
| Observations | 90,578 | 90,578 | 90,578 | 90,578 | 90,578 | 90,578 | 90,578 | 90,578 |
| R-squared | 0.068 | 0.068 | 0.068 | 0.068 | 0.068 | 0.068 | 0.068 | 0.068 |

Notes: ***Significant at 1%, **Significant at 5%, *Significant at 10 %. Prefecture fixed effects, year fixed effects and Province year time trend are included. Standard errors clustered by county in parentheses, unless otherwise indicated. Column (1) to column (6) include control variables proxied with the prenatal examination like whether the respondent mother received pre-natal doctor care and whether the child is delivered at home, hospital or clinic is assisted by doctor, nurse or midwife. County baseline controls include grain production, school number, teacher number, GDP, industry ratio, service ratio, family savings, fixed asset, government expenditure and population density. The weight is calculated as follows: First, determine the number of women in different age groups for each year in the sample regions. Next, using the age-group-specific female population data from the 1953, 1964, and 1982 Censuses, divide the sample number of women in each age group by the corresponding female population in the Census data for that year. The weight for each age group is then obtained by taking the reciprocal of this ratio.

Table S19. Heterogeneous effects: The effects of HYVs on the infant mortality using rural sample

|  | Whether infant died (1=yes, 0=no) | | | | | | |
| --- | --- | --- | --- | --- | --- | --- | --- |
| Panel a | (1) | | (2) | | (3) | | (4) |
| Lnyield_aver (t-3~t-1)***suit**aver | -0.168 | |  | |  | |  |
|  | (0.107) | |  | |  | |  |
| Lnyield_aver (t-3~t-1)***suit**aver*Infant gender (Male = 1) | -0.087** | |  | |  | |  |
|  | (0.038) | |  | |  | |  |
| Lnyield_wheat (t-3~t-1)***suit**wt |  | | -0.254*** | |  | | -0.241** |
|  |  | | (0.093) | |  | | (0.101) |
| Lnyield_wheat (t-3~t-1)***suit**wt* Infant gender (Male = 1) |  | | -0.071** | |  | | -0.065* |
|  |  | | (0.029) | |  | | (0.034) |
| Lnyield_maize (t-3~t-1)***suit**mz |  | |  | | -0.057 | | -0.031 |
|  |  | |  | | (0.078) | | (0.081) |
| Lnyield_maize (t-3~t-1)***suit**mz* Infant gender (Male = 1) |  | |  | | -0.048 | | -0.012 |
|  |  | |  | | (0.032) | | (0.036) |
| Observations | 65,362 | | 65,362 | | 65,362 | | 65,362 |
| R-squared | 0.042 | | 0.042 | | 0.042 | | 0.042 |
| Panel b |  | | | | | | |
| Lnyield_aver (t-3~t-1)***suit**aver | -0.315* | |  | |  | |  |
|  | (0.189) | |  | |  | |  |
| Lnyield_aver (t-3~t-1)***suit**aver*Birth control (Complemented = 1) | 0.116 | |  | |  | |  |
|  | (0.155) | |  | |  | |  |
| Lnyield_wheat (t-3~t-1)***suit**wt |  | | -0.306* | |  | | -0.276* |
|  |  | | (0.167) | |  | | (0.164) |
| Lnyield_wheat (t-3~t-1)***suit**wt*Birth control (Complemented = 1) |  | | 0.017 | |  | | 0.002 |
|  |  | | (0.169) | |  | | (0.169) |
| Lnyield_maize (t-3~t-1)***suit**mz |  | |  | | -0.138 | | -0.088 |
|  |  | |  | | (0.121) | | (0.116) |
| Lnyield_maize (t-3~t-1)***suit**mz*Birth control (Complemented = 1) |  | |  | | 0.063 | | 0.057 |
|  |  | |  | | (0.092) | | (0.090) |
| Observations | 65,362 | | 65,362 | | 65,362 | | 65,362 |
| R-squared | 0.042 | | 0.042 | | 0.042 | | 0.042 |
| Panel c |  | | | | | | |
| Lnyield_aver (t-3~t-1)***suit**aver | -0.217** | |  | |  | |  |
|  | (0.109) | |  | |  | |  |
| Lnyield_aver (t-3~t-1)***suit**aver*Mother age (≥35 = 1) | 0.080 | |  | |  | |  |
|  | (0.143) | |  | |  | |  |
| Lnyield_wheat (t-3~t-1)***suit**wt |  | | -0.298*** | |  | | -0.284*** |
|  |  | | (0.094) | |  | | (0.101) |
| Lnyield_wheat (t-3~t-1)***suit**wt*Mother age (≥35 = 1) |  | | 0.147 | |  | | 0.212 |
|  |  | | (0.111) | |  | | (0.134) |
| Lnyield_maize (t-3~t-1)***suit**mz |  | |  | | -0.079 | | -0.029 |
|  |  | |  | | (0.080) | | (0.083) |
| Lnyield_maize (t-3~t-1)***suit**mz*Mother age (≥35 = 1) |  | |  | | -0.079 | | -0.186 |
|  |  | |  | | (0.137) | | (0.165) |
| Observations | 65,362 | | 65,362 | | 65,362 | | 65,362 |
| R-squared | 0.042 | | 0.042 | | 0.042 | | 0.042 |
| Panel d |  | | | | | | |
| Lnyield_aver (t-3~t-1)***suit**aver | -0.214* | |  | |  | |  |
|  | (0.108) | |  | |  | |  |
| Lnyield_aver (t-3~t-1)***suit**aver*Mother edu (High school = 1) | -0.430 | |  | |  | |  |
|  | (0.370) | |  | |  | |  |
| Lnyield_wheat (t-3~t-1)***suit**wt |  | | -0.291*** | |  | | -0.275*** |
|  |  | | (0.093) | |  | | (0.100) |
| Lnyield_wheat (t-3~t-1)***suit**wt*Mother edu (High school = 1) |  | | -0.105 | |  | | 0.856 |
|  |  | | (0.442) | |  | | (0.712) |
| Lnyield_maize (t-3~t-1)***suit**mz |  | |  | | -0.081 | | -0.036 |
|  |  | |  | | (0.078) | | (0.080) |
| Lnyield_maize (t-3~t-1)***suit**mz*Mother edu (High school = 1) |  | |  | | -0.700*** | | -1.320** |
|  |  | |  | | (0.267) | | (0.656) |
| Controls | Yes | | Yes | | Yes | | Yes |
| County baseline*Time Trend | Yes | | Yes | | Yes | | Yes |
| Prefecture FE | Yes | | Yes | | Yes | | Yes |
| Year FE | Yes | | Yes | | Yes | | Yes |
| Province year FE | Yes | | Yes | | Yes | | Yes |
| Observations | 65,362 | 65,362 | | 65,362 | | 65,362 | |
| R-squared | 0.042 | 0.042 | | 0.042 | | 0.042 | |

Notes: ***Significant at 1%, **Significant at 5%, *Significant at 10 %. Prefecture fixed effects, year fixed effects and Province year fixed effects are included. Standard errors clustered by county in parentheses, unless otherwise indicated.

Table S20. Heterogeneous effects using the number of the HYVs variables using rural sample as a robustness check

|  | Whether infant died (1=yes, 0=no) | | | |
| --- | --- | --- | --- | --- |
| Panel a | (1) | (2) | (3) | (4) |
| Amount (t-3~t-1)***suit**aver | -0.083 |  |  |  |
|  | (0.078) |  |  |  |
| Amount (t-3~t-1)***suit**aver*Infant gender (Male = 1) | -0.055* |  |  |  |
|  | (0.032) |  |  |  |
| Num_wheat (t-3~t-1)***suit**wt |  | -0.166 |  | -0.142 |
|  |  | (0.103) |  | (0.105) |
| Num_wheat (t-3~t-1)***suit**wt*Infant gender (Male = 1) |  | -0.059* |  | -0.069* |
|  |  | (0.035) |  | (0.040) |
| Num_maize (t-3~t-1)***suit**mz |  |  | -0.120 | -0.129 |
|  |  |  | (0.127) | (0.126) |
| Num_maize (t-3~t-1)***suit**mz*Infant gender (Male = 1) |  |  | -0.040 | 0.061 |
|  |  |  | (0.100) | (0.114) |
| Observations | 65,362 | 65,362 | 65,362 | 65,362 |
| R-squared | 0.042 | 0.042 | 0.042 | 0.042 |
| Panel b |  | | | |
| Amount (t-3~t-1)***suit**aver | -0.055 |  |  |  |
|  | (0.101) |  |  |  |
| Amount (t-3~t-1)***suit**aver*Birth control (Complemented = 1) | -0.085 |  |  |  |
|  | (0.101) |  |  |  |
| Num_wheat (t-3~t-1)***suit**wt |  | -0.069 |  | -0.050 |
|  |  | (0.172) |  | (0.179) |
| Num_wheat (t-3~t-1)***suit**wt*Birth control (Complemented = 1) |  | -0.169 |  | -0.165 |
|  |  | (0.180) |  | (0.187) |
| Num_maize (t-3~t-1)***suit**mz |  |  | -0.070 | -0.058 |
|  |  |  | (0.126) | (0.130) |
| Num_maize (t-3~t-1)***suit**mz*Birth control (Complemented = 1) |  |  | -0.140 | -0.093 |
|  |  |  | (0.155) | (0.157) |
| Observations | 65,362 | 65,362 | 65,362 | 65,362 |
| R-squared | 0.042 | 0.042 | 0.042 | 0.042 |
| Panel c |  | | | |
| Amount (t-3~t-1)***suit**aver | -0.113 |  |  |  |
|  | (0.079) |  |  |  |
| Amount (t-3~t-1)***suit**aver*Mother age (≥35 = 1) | 0.007 |  |  |  |
|  | (0.088) |  |  |  |
| Num_wheat (t-3~t-1)***suit**wt |  | -0.204* |  | -0.185* |
|  |  | (0.106) |  | (0.107) |
| Num_wheat (t-3~t-1)***suit**wt*Mother age (≥35 = 1) |  | 0.086 |  | 0.109 |
|  |  | (0.111) |  | (0.138) |
| Num_maize (t-3~t-1)***suit**mz |  |  | -0.143 | -0.092 |
|  |  |  | (0.127) | (0.129) |
| Num_maize (t-3~t-1)***suit**mz*Mother age (≥35 = 1) |  |  | 0.031 | -0.109 |
|  |  |  | (0.222) | (0.293) |
| Observations | 65,362 | 65,362 | 65,362 | 65,362 |
| R-squared | 0.042 | 0.042 | 0.042 | 0.042 |
| Panel d |  | | | |
| Amount (t-3~t-1)***suit**aver | -0.111 |  |  |  |
|  | (0.078) |  |  |  |
| Amount (t-3~t-1)***suit**aver*Mother edu (High school = 1) | -0.407* |  |  |  |
|  | (0.242) |  |  |  |
| Num_wheat (t-3~t-1)***suit**wt |  | -0.198* |  | -0.179* |
|  |  | (0.105) |  | (0.106) |
| Num_wheat (t-3~t-1)***suit**wt*Mother edu (High school = 1) |  | -0.494 |  | -0.540 |
|  |  | (0.310) |  | (0.442) |
| Num_maize (t-3~t-1)***suit**mz |  |  | -0.140 | -0.095 |
|  |  |  | (0.124) | (0.124) |
| Num_maize (t-3~t-1)***suit**mz*Mother edu (High school = 1) |  |  | -0.535 | 0.181 |
|  |  |  | (0.403) | (0.696) |
| Controls | Yes | Yes | Yes | Yes |
| County baseline*Time Trend | Yes | Yes | Yes | Yes |
| Prefecture FE | Yes | Yes | Yes | Yes |
| Year FE | Yes | Yes | Yes | Yes |
| Province year FE | Yes | Yes | Yes | Yes |
| Observations | 65,362 | 65,362 | 65,362 | 65,362 |
| R-squared | 0.042 | 0.042 | 0.042 | 0.042 |

Notes: ***Significant at 1%, **Significant at 5%, *Significant at 10 %. Prefecture fixed effects, year fixed effects and Province year fixed effects are included. Standard errors clustered by county in parentheses, unless otherwise indicated.
